# A Toolkit and Robust Pipeline for the Generation of Fosmid-Based Reporter Genes in *C. elegans*

**DOI:** 10.1371/journal.pone.0004625

**Published:** 2009-03-04

**Authors:** Baris Tursun, Luisa Cochella, Inés Carrera, Oliver Hobert

**Affiliations:** Howard Hughes Medical Institute, Department of Biochemistry and Molecular Biophysics, Columbia University Medical Center, New York, New York, United States of America; Massachusetts General Hospital/Harvard Medical School, United States of America

## Abstract

Engineering fluorescent proteins into large genomic clones, contained within BACs or fosmid vectors, is a tool to visualize and study spatiotemporal gene expression patterns in transgenic animals. Because these reporters cover large genomic regions, they most likely capture all *cis*-regulatory information and can therefore be expected to recapitulate all aspects of endogenous gene expression. Inserting tags at the target gene locus contained within genomic clones by homologous recombination (“recombineering”) represents the most straightforward method to generate these reporters. In this methodology paper, we describe a simple and robust pipeline for recombineering of fosmids, which we apply to generate reporter constructs in the nematode *C. elegans*, whose genome is almost entirely covered in an available fosmid library. We have generated a toolkit that allows for insertion of fluorescent proteins (GFP, YFP, CFP, VENUS, mCherry) and affinity tags at specific target sites within fosmid clones in a virtually seamless manner. Our new pipeline is less complex and, in our hands, works more robustly than previously described recombineering strategies to generate reporter fusions for *C. elegans* expression studies. Furthermore, our toolkit provides a novel recombineering cassette which inserts a SL2-spliced intercistronic region between the gene of interest and the fluorescent protein, thus creating a reporter controlled by all 5′ and 3′ *cis*-acting regulatory elements of the examined gene without the direct translational fusion between the two. With this configuration, the onset of expression and tissue specificity of secreted, sub-cellular compartmentalized or short-lived gene products can be easily detected. We describe other applications of fosmid recombineering as well. The simplicity, speed and robustness of the recombineering pipeline described here should prompt the routine use of this strategy for expression studies in *C. elegans*.

## Introduction

Knowing the expression pattern of a gene is essential for the complete understanding of its function. In essentially every experimental system - including our system of choice, the nematode *C. elegans* - the ultimate product of gene expression can be directly visualized in the form of a fluorescent-protein reporter, such as GFP, attached to the gene of interest [1]. A key issue in generating reporter gene fusions is that, in order to accurately reflect all aspects of the temporal and spatial pattern of expression of the gene of interest, as many *cis*-regulatory control regions as possible need to be included in the DNA construct. However, quite often, expression patterns are inferred from “promoter fusions” in which only a few kilobases of sequence upstream of the predicted start of the gene are fused to *gfp* [2]. However, it has become increasingly clear that regulatory information that affects the expression pattern of a gene can be found in every part of the respective locus, such as within introns or downstream of a gene (e.g. [3]). The recently uncovered pervasiveness of gene regulation by miRNAs, small regulatory RNAs that regulate gene expression post-transcriptionally [4], also highlights the importance of including 3′UTR sequences in reporter gene constructs. Other aspects of posttranscriptional gene regulation at the level of alternative splicing, mRNA localization, or protein degradation are determined by elements in the coding region as well as in 5′ and 3′ flanking sequences. These issues highlight the need for an accurate reporter to include as much of the gene coding sequence and the 5′ and 3′ flanking sequences as possible. Conventional methods, such as subcloning the genomic locus in a standard set of vectors [5] or fusing the genomic locus to the reporter gene by PCR [6] have limitations in terms of the size of the DNA that can be included in a reporter. The recombination-mediated engineering of fluorescent protein fusions in the context of genomic clones contained in BACs or fosmids is therefore emerging as a tool to study gene expression that offers the best compromise between facility in generation and completeness of *cis*-regulatory information [7], [8].

In *C. elegans*, a readily available fosmid library (http://elegans.bcgsc.bc.ca/) that covers ∼80% of the genome and ∼90% of the worm genes (D. Moerman, personal communication) is often used for complementation of mutant phenotypes both during the identification of new mutants and the subsequent analysis of cloned mutants. Fosmids usually contain 35–40 Kbp genomic fragments and because of the >5X clone coverage, one can usually find a fosmid where the gene of interest is close to the center, with at least 2–3 genetic loci on either side and therefore likely containing all *cis*-regulatory control regions. This fosmid library provides an excellent starting point for the construction of reporter genes and it is what we use for the reporter tagging procedure we present here. Engineering fluorescently labeled genes of interest in a fosmid context also potentially allows for evaluation of the functionality of the tagged gene as it should rescue the mutant phenotype of the gene under study.

Recombineering a reporter gene into a fosmid relies on homologous recombination in bacteria expressing the λ Red recombinase [9]. In brief, a linear DNA fragment containing the tag of interest flanked by ∼50 bp of sequence on both sides of the site of insertion is introduced in a bacterial strain containing the fosmid (or BAC) of choice and the λ Red recombinase. The recombination reaction will result in the seamless insertion of the tag into the desired position in the vector. However, because the reaction is not sufficiently efficient, insertion of a non-selectable tag has to be done in a two-step procedure.

**Figure 1:**
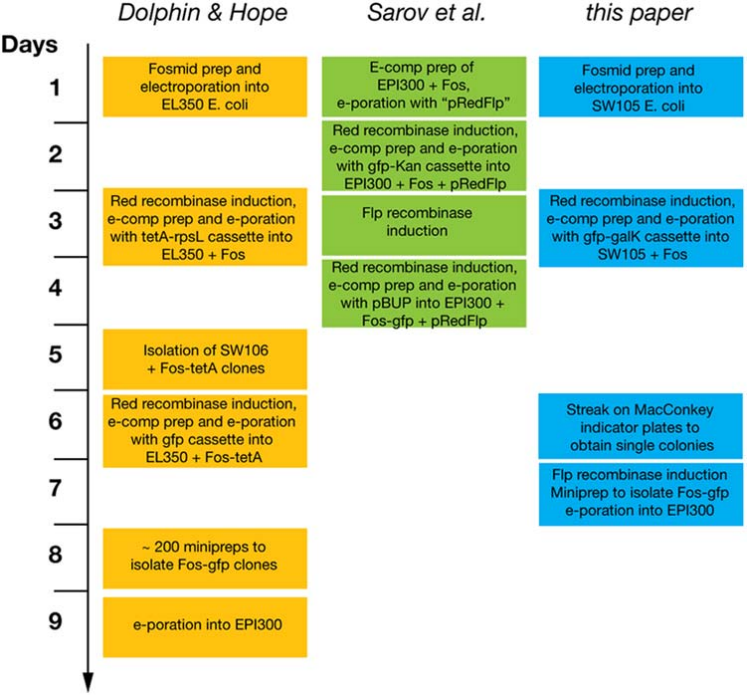
Timeline comparison of available recombineering protocols.

**Figure 2:**
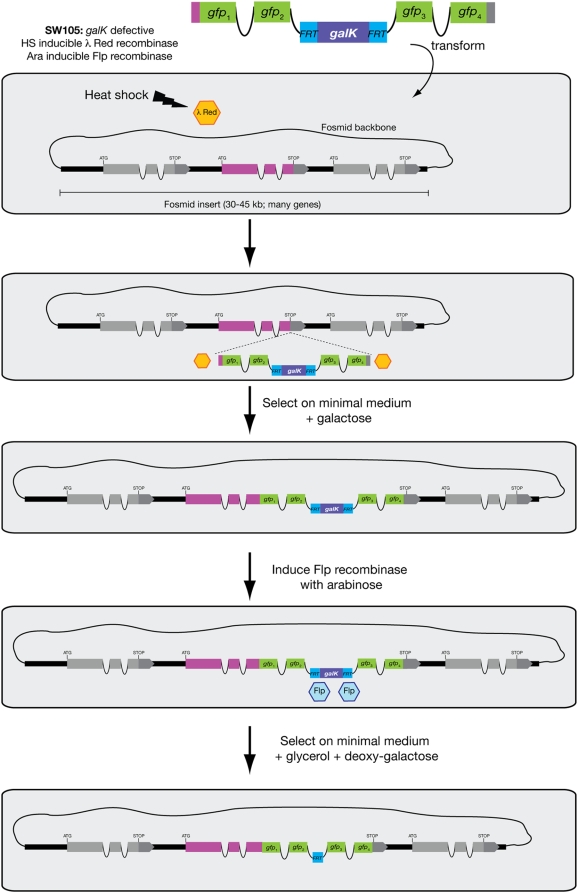
Recombineering pipeline. A cassette containing the tag of interest and the *FRT-galK-FRT* (*FgF*) selection module, and flanked by sequence homologous to the desired insertion site can be recombined into the gene of choice in the context of a fosmid genomic clone. Recombination is carried out in SW105, an *E. coli* strain with heat inducible λ Red recombinase. Recombinant clones are selected on minimal medium with galactose. Excision of the *FgF* selection module is carried out by Flp recombinase, which is induced by addition of arabinose. Excision is typically complete, but to aid in the isolation of pure recombineered fosmids selection for loss of *galK* can be done in medium containing deoxy-galactose. Note that the *FRT* “scar” left after *galK* excision resides in an intron and therefore has no impact on protein coding sequences. Also note that the cassette can be inserted anywhere.

Two recent protocols established fosmid recombineering as a tool tailored to analyze gene expression in *C. elegans*, providing an important advance compared to conventional reporter gene approaches [10], [11]. In the protocol developed by Dolphin and Hope, a selectable marker is inserted in the position of interest allowing for selection of the rare recombination event [11](Fig. 1 and 2). Then, in a similar recombination reaction the first marker is removed and replaced by the tag of interest with the aid of a counter-selectable marker. In our hands this method has proven quite inefficient. In contrast to the protocol that we describe here, which we never found to fail, the Dolphin & Hope protocol has very variable efficiency, that is, in our hands, it sometimes worked well, other times it barely worked or did not work at all (see Supplement Table S1 for a comparison). We ascribe these difficulties to the high level of background (false positives) that arises from the selection for loss of a counter-selectable marker. Such a loss will not only occur upon the desired recombination event but can also be simply caused by mutations that disrupt the function of the selectable marker. The substantial number of false positive colonies resulting from this second selection step, necessitates the screening of up to hundreds of colonies before obtaining the correctly recombineered end-product (Supplement Table S1).

Some improvement of this original protocol has been reported by Sarov et al., who described a strategy involving two recombinases to engineer BACs [10](Fig. 1). First, using λ Red recombinase, a cassette containing both the tag of interest and a selectable marker flanked by *FRT*/Flp sites is inserted by homologous recombination at the position of interest. Then, using Flp recombinase the selectable marker is excised, leaving behind the tag of interest and a 34 bp “scar” corresponding to one of the *FRT* sites. While this technique indeed seems to work efficiently, the published protocol is somewhat complicated and requires additional plasmids, selection media and reagents. The protocol that we describe here makes use of a commonly available bacterial strain, SW105, and a single selection marker, the *galK* gene, which allows for very efficient, both positive and negative selection [12], with the negative selection allowing to confirm the flip-out event. The Sarov protocol does not offer negative selection to aid in the excision of the selection marker from BACs. This is problematic as we have encountered incomplete excision in a few cases, and thus the availability of a counter-selectable marker can significantly simplify the isolation of the desired recombineered clone.

A timeline comparison of the described methods is shown in Fig. 1 and efficiency comparisons are shown in Supplement Table S1. The protocol that we describe in this paper takes 2 days longer than that established by Sarov et al., but we believe that the simplicity and robustness of our strategy compensate for this.

As another improvement to the previously published protocols [10], [11], we have designed the recombineering cassettes such that the *FRT* “scar” is hidden in one of the artificial introns of the inserted fluorescent protein, resulting in a virtually seamless insertion of the tag of interest. We have also generated bicistronic reporter cassettes in which the gene of interest becomes separated from the reporter gene. We have constructed a set of cassettes containing a battery of distinct fluorescent tags and the *galK* gene flanked by *FRT* sites in multiple configurations, such that any researcher could order one pair of primers, a bacterial strain and minimal medium with galactose to obtain a recombineered fosmid in one week. We also discuss other applications based on this pipeline, such as the alterations of the length of fosmid inserts or replacement of specific sequences within fosmids.

## Methods

### Design of Pipeline and Toolkit

#### Overview

An overview of the design of our pipeline is shown in Fig. 2. The first step to generate a reporter containing the genomic context of the gene of interest - and thus, likely most *cis*-regulatory information for its expression – is to choose an appropriate genomic clone. In the case of *C. elegans*, a fosmid library containing ∼90% of the worm genes with >5X coverage is readily available (http://www.geneservice.co.uk/products/clones/Celegans_Fos.jsp) and perfectly suited for recombineering. Typically, a fosmid where the gene of interest is close to the center of the insert, with >10 Kbp of flanking sequence on each side can be found (examples are shown in the “Example Applications” section below). If this is not the case, the strategy we present could also be used to extend the genomic insert contained in the fosmid.

For recombineering purposes the acceptable vector backbones are those that contain single copy origins of replication such as BACs and fosmids as otherwise, in the presence of multiple vector copies, recombination of only one or a few DNA molecules will result in mixed vector populations in a single bacterium. While the fosmids are typically supplied in the *E. coli* strain EPI300 for proper maintenance, for the recombination procedure they need to be isolated and transformed via electroporation into strain SW105, which contains both required recombinases under the control of independent, inducible promoters [12].

Once the fosmid of interest is in SW105, the bacteria are heat shocked to induce expression of the λ Red recombinase and then washed for electroporation (see detailed protocol below). The electrocompetent cells containing the fosmid of interest and the recombinase are then ready to take up the linear DNA product carrying the tag of interest for recombination (Fig. 2).

**Figure 3:**
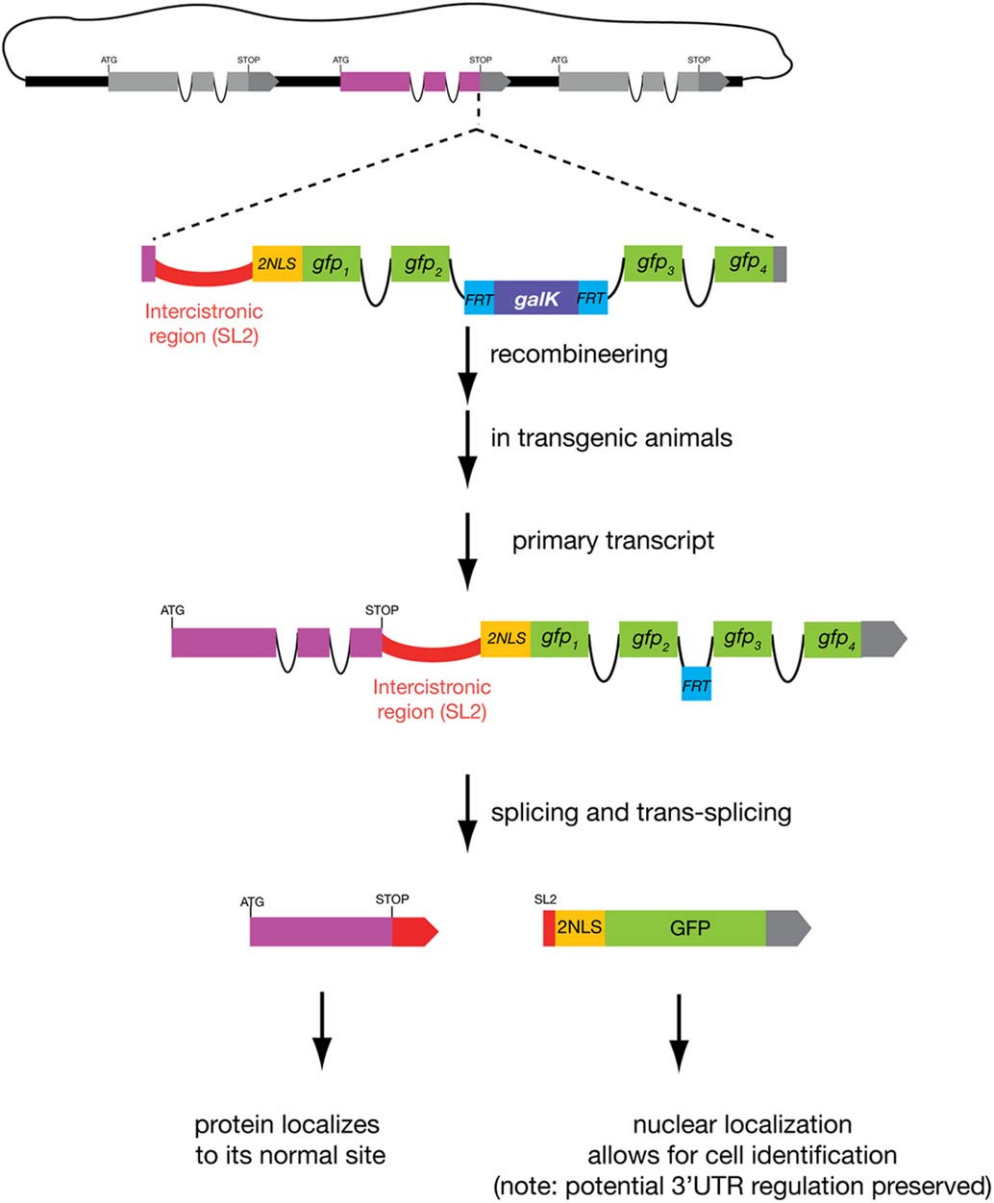
A bicistronic recombineering cassette. This series of cassettes can be inserted between the stop codon and the 3′ UTR of the gene of interest. The recombination procedure is identical to that described for other cassettes but the end result is an operon-like configuration that after splicing and trans-splicing will give rise to two gene products: an unmodified protein of interest and a nuclear-localized fluorescent protein that reports the onset of expression and cellular focus of action of the protein of interest. Note that the endogenous protein loses its 3′ UTR but this remains on the reporter cistron and thus potential 3′ UTR-mediated gene regulation should be preserved. These cassettes are of particular use for subcellularly-compartmentalized or secreted proteins, or proteins with very short half-lives.

The reporter tag of choice is introduced in the form of a cassette also containing the selectable marker *galK* flanked by *FRT* sites, which will later be targeted by the Flp recombinase (the *FRT-galK-FRT* module or *FgF*)(Fig. 3 & Table 1). The whole cassette is PCR amplified from a vector template (Table 1) with specific oligonucleotides that add ∼50 bp of sequence homologous to each side of the desired site of insertion in the gene of choice. When transformed into bacteria containing the target fosmid and λ Red recombinase, homologous recombination between the ends of the PCR cassette and the fosmid will occur. The SW105 *E. coli* strain is defective for the galactokinase activity provided by *galK* and cannot grow on minimal medium with galactose as the sole carbon source. Thus, recombinants stably maintaining the *galK*-containing cassette can be selected for by growth on minimal medium with galactose. We have chosen *galK* rather than other markers, such as those conferring antibiotic resistance used in previous *C. elegans* recombineering protocols [10], [11], because *galK* allows for both positive and negative selection on the same gene: when supplied with deoxy-galactose, bacteria containing the *galK* gene will phosphorylate it to a toxic intermediate and die. The efficiency of selection with *galK* is much better than selection with previously reported schemes [12].

**Table 1:** Fosmid recombineering cassettes.

Cassettes (*FgF = FRT-galK-FRT*)	Plasmid name	Properties	5′ sequence (X_50_ = gene specific sequence)	3′sequence (X_50_ = gene specific sequence)
**GFPint-** ***FgF***	pBALU1	*FRT-galK-FRT* in the 2^nd^ intron of *gfp*		
		N-terminal fusion	5′ X_50_-atgagtaaaggagaagaacttttcac	5′ catggcatggatgaactatacaaa-ATGX_50_ 3′
		C-terminal fusion	5′ X_50_-atgagtaaaggagaagaacttttcac	5′ catggcatggatgaactatacaaa-STOPX_50_ 3′
**YFPint-** ***FgF***	pBALU2	as above but with *yfp*		
		N-terminal fusion	5′ X_50_-atgagtaaaggagaagaacttttcac	5′ catggcatggatgaactatacaaa-ATGX_50_ 3′
		C-terminal fusion	5′ X_50_-atgagtaaaggagaagaacttttcac	5′ catggcatggatgaactatacaaa-STOPX_50_ 3′
**CFPint-** ***FgF***	pBALU3	as above but with c*fp*		
		N-terminal fusion	5′ X_50_-atgagtaaaggagaagaacttttcac	5′ catggcatggatgaactatacaaa-ATGX_50_
		C-terminal fusion	5′ X_50_-atgagtaaaggagaagaacttttcac	3′ catggcatggatgaactatacaaa-STOPX_50_ 3′
**mChOint-** ***FgF***	pBALU4	as above but with *mChOpti*		
		N-terminal fusion	5′ X_50_-atggtctcaaagggtgaagaagataac	5′ ggtggcatggatgaattgtataag-ATGX_50_ 3′
		C-terminal fusion	5′ X_50_-atggtctcaaagggtgaagaagataac	5′ ggtggcatggatgaattgtataag-STOPX_50_ 3′
***FgF*** **-2xFLAG-Venus**	pBALU5	*FRT-galK-FRT* in frame w/ 2xFLAG; no introns; C-terminal fusions	5′ X_50_-gggaagttcctatactttctagagaataggaacttccctgttgac 3′	5′ ctcggcatggacgagctgtacaag-STOPX_50_ 3′
**Venus-** ***FgF***	pBALU6	Venus in frame w/ *FRT-galK-FRT*; no introns; N-terminal fusion	5′ X_50_-atggtgagcaagggcgaggagctgttc 3′	5′ gcggccgcgaagttcctatactttctagagaataggaacttc-ATGX_50_ 3′
***FgF*** **-mCherry**	pBALU7	*FRT-galK-FRT* in frame w/ mCherry; no introns; C-terminal fusions	5′ X_50_-gggaagttcctatactttctagagaataggaacttccctgttgac 3′	5′ ggcggcatggacgagctgtacaag-STOPX_50_ 3′
**mCherry-** ***FgF***	pBALU8 pBALU8*	*as above but for N-terminal fusion*	5′ X_50_-atggtgagcaagggcgaggaggataac 3′	5′ gcggccgcgaagttcctatactttctagagaataggaacttc-ATGX_50_ 3′
**gSL2-NLS-GFPint-** ***FgF***	pBALU9	bicistronic; *FRT-galK-FRT* in 2^nd^ GFP intron; use C-term after STOP	5′ X_50_STOP-gctgtctcatcctactttcacctagttaac 3′	5′ catggcatggatgaactatacaaatag-X_50_ 3′
**gSL2-NLS-YFPint-** ***FgF***	pBALU10 pBALU10*	*as above but with yfp*	5′ X_50_STOP-gctgtctcatcctactttcacctagttaac 3′	5′ catggcatggatgaactatacaaatag-X_50_ 3′
**gSL2-NLS-CFPint-** ***FgF***	pBALU11	*as above but with cfp*	5′ X_50_STOP-gctgtctcatcctactttcacctagttaac 3′	5′ catggcatggatgaactatacaaatag-X_50_ 3′
**gSL2-NLS-mChOint-** ***FgF***	pBALU12	*as above but with mChOpti*	5′ X_50_STOP-gctgtctcatcctactttcacctagttaac 3′	5′ ggtggcatggatgaattgtataagtaa-X_50_ 3′
**gSL2-NLS-** ***FgF*** **-FLAG-Venus**	pBALU13	bicistronic; *FRT* in frame w/ 2xFLAG; no introns; use C-term after STOP	5′ X_50_STOP-gctgtctcatcctactttcacctagttaac 3′	5′ ctcggcatggacgagctgtacaagtaa-X_50_ 3′
**gSL2-NLS-** ***FgF*** **-mCherry**	pBALU14	bi-*cis*tronic; *FRT* in frame w/ mCherry no introns; use C-term after STOP	5′ X_50_STOP-gctgtctcatcctactttcacctagttaac 3′	5′ ggcggcatggacgagctgtacaagtaa-X_50_ 3′
**rSL2-NLS-GFPint-** ***FgF***	pBALU15	bicistronic; *FRT-galK-FRT* in 2^nd^ GFP intron; use C-term after STOP	5′ X_50_STOP-gccatgttgttaccttgtattaaaacaatg 3′	5′ catggcatggatgaactatacaaatag-X_50_ 3′
**rSL2-NLS-YFPint-** ***FgF***	pBALU16 pBALU16*	*as above but with yfp*	5′ X_50_STOP-gccatgttgttaccttgtattaaaacaatg 3′	5′ catggcatggatgaactatacaaatag-X_50_ 3′
**rSL2-NLS-CFPint-** ***FgF***	pBALU17 pBALU17*	*as above but with cfp*	5′ X_50_STOP-gccatgttgttaccttgtattaaaacaatg 3′	5′ catggcatggatgaactatacaaatag-X_50_ 3′
**rSL2-NLS-mChOint-** ***FgF***	pBALU18 pBALU18*	*as above but with mChOpti*	5′ X_50_STOP-gccatgttgttaccttgtattaaaacaatg 3′	5′ ggtggcatggatgaattgtataagtaa-X_50_ 3′
**rSL2-NLS-** ***FgF*** **-FLAG-Venus**	pBALU19	bicistronic; *FRT* in frame w/ 2xFLAG; no introns; use C-term after STOP	5′ X_50_STOP-gccatgttgttaccttgtattaaaacaatg 3′	5′ ctcggcatggacgagctgtacaagtaa-X_50_ 3′
**rSL2-NLS-** ***FgF*** **-mCherry**	pBALU20	bicistronic; *FRT* in frame w/ mCherry no introns; use C-term after STOP	5′ X_50_STOP-gccatgttgttaccttgtattaaaacaatg 3′	5′ ggcggcatggacgagctgtacaagtaa-X_50_ 3′
***unc-54*** ** 3′UTR +** ***FgF***	pBALU21	replaces endogenous 3′UTR; use C-term after STOP	5′ X_50_ gtccaattactcttcaacatccctacatgc 3′	5′ gcggccgcgaagttcctatactttctagagaatggaacttc-STOPX_50_ 3′
**kanamycin gene +pCC1FOS homology**	pBALU-ext	extension at 5′ end: PCR fuse the cassette to the 3′ end of KAN gene:	5′ gccagggttttcccagtcacgac 3′	5′ gttcttctgaattgaaaaaggaagagt-X_30_ 3′
		extension at 3′ end: PCR fuse the cassette to the 5′ end of KAN gene:	5′ X_30_-ccggaattgccagctggggcgccctc 3′	5′ gtaatcatggtcatagctgtttcctgtg 3′

The vector backbone for the pBALU vectors is the pCR TOPO cloning vector (Invitrogen). For complete plasmid sequences, see Supplement Text S1.

Note that the intronless cassettes are specifically required to allow for double-recombineering with the intron-containing reporter genes. If there were overlap between the sequentially introduced reporters, undesired homologous recombination could occur. STOP represents the stop codon from the gene of interest. mChO (mChOpti) is an intron containing, and *C. elegans* codon usage optimized mCherry variant as described in McNally et al., 2006. For each vector series (e.g. pBALU15-18), at least one, if not all, color version was explicitly tested in recombineering reactions. The pBALU vectors that are also available with modified *FRTs* for double recombineering are labeled pBALUxx*.

The desired end product should only contain the fluorescent protein or tag of choice fused to the gene of interest, so after selection of the rare recombination event the selectable marker must be removed. This is accomplished by inducing the second recombinase, Flipase (Flp), which is under control of the arabinose operator. Addition of arabinose will induce expression of the Flp recombinase, which will excise the marker flanked by the *FRT* sites. This reaction will leave behind a 34 bp “scar” corresponding to one of the *FRT* sites. This scar is the price to pay for a much improved efficiency in the correct removal of the counter-selectable marker compared to the original Dolphin and Hope protocol (Supplement Table S1). To eliminate any possible impact of the scar on the resulting fusion protein, we designed a cassette that hides the scar in one of the introns of the fluorescent tag. For this purpose we have inserted the *FRT-galK-FRT (“FgF”)* module in the second intron of four different fluorescent reporter proteins (“FPs”), GFP, YFP, CFP and an intron-containing mCherry, mChOpti [13](Fig. 3; Table 1; Supplement Text S1). These cassettes can be used for fusion of the fluorescent proteins to either the N- or C-terminus of the protein of interest without adding any extra amino acids to the translated fused product. We have also designed intronless fluorescent reporters (some with FLAG-type affinity tags for antibody recognition) in which the scar is translated as an 8-amino acid linker between the reporter and the gene of interest (Fig. 3; Table 1). Such cassettes were designed for both N- or C-terminal fusions (Fig. 3; Table 1).

#### Cassette vectors

Our cassette-containing vectors are called “pBALU”. In contrast to previous protocols we have kept our cassettes in the TOPO based backbones (Invitrogen) rather than cloning them into a plasmid backbone that cannot replicate in SW105. We have never experienced any background problems due to transformation with traces of the pBALU vectors used as templates for amplifying the recombineering cassettes, since the PCR products are always gel purified. If, for some reason, gel purification of the PCR reaction is not desired, most cassettes can be simply isolated with high yield from the TOPO based pBALU backbone by EcoRI digestion. The purified fragment serves then as a safe template for all future PCR reactions. The high yield and easy manipulation of TOPO-based vectors allows for simple modification of our cassettes and permits the generation of new ones.

#### Bicistronic cassettes

In addition to a set of standard cassettes (Table 1), we have generated a set of cassettes that should prove useful for studying the expression of proteins that are secreted, contain transmembrane domains or localize to other specialized subcellular compartments. On the one hand, direct reporter fusions to this sort of proteins reveal their authentic localization (allowing also to visualize dynamics in their localization patterns). On the other hand, specialized subcellular localization patterns (or secretion) can make the identification of the cell types in which these genes are expressed, exceedingly difficult - especially because cellular identification in *C. elegans* usually takes advantage of the defined positions of individual nuclei or of specific cellular shapes. To circumvent this problem, we adopted a strategy in which the gene of interest is fused to a fluorescent reporter in a bicistronic configuration (Fig. 3) [14]. Cleavage and trans-splicing of this bicistronic primary transcript to SL2 will result in the production of two mRNA cistrons, the first one encoding the endogenous, unmodified protein and the second one encoding the fluorescent reporter [15], [16]. While the reporter monitors gene-expression regulation of the entire locus, the resulting fluorescent protein is not physically attached to the protein of interest. Instead, the fluorescent protein is targeted to the nucleus via an appended nuclear localization signal (NLS). Nuclear fluorescence then allows for easy cell identification. This approach can also be used to study the onset of expression of proteins that are intrinsically very unstable.

To generate such fusions by recombineering, our cassettes contain the intercistronic region of either one of two different *C. elegans* operons [15], [16], followed by a NLS-fluorescent protein-encoding gene and the *FRT-galK-FRT* module for selection (Fig. 3). We provide two separate intercistronic cassettes so as to allow for two independent (and non-interfering) recombinations into one fosmid (if the same intercistronic region were used, sequence homology could potentially interfere with the second recombination). These cassettes should be inserted immediately after the stop codon of the gene of interest. Again, for all intron-containing FPs we have inserted the *FRT-galK-FRT* module in the intron, while for those that have no intron and thus will retain the *FRT* “scar” within the coding sequence, we have designed the cassettes in such a way that the scar is translated as a linker between the NLS and the fluorescent protein. The procedure for amplification and insertion of the cassette and further excision of the *FRT*-galK fragment is essentially identical to that described for the other cassettes, with the only difference that the cassette should be inserted after the endogenous stop codon.

#### Double-labeling cassettes

**Figure 4:**
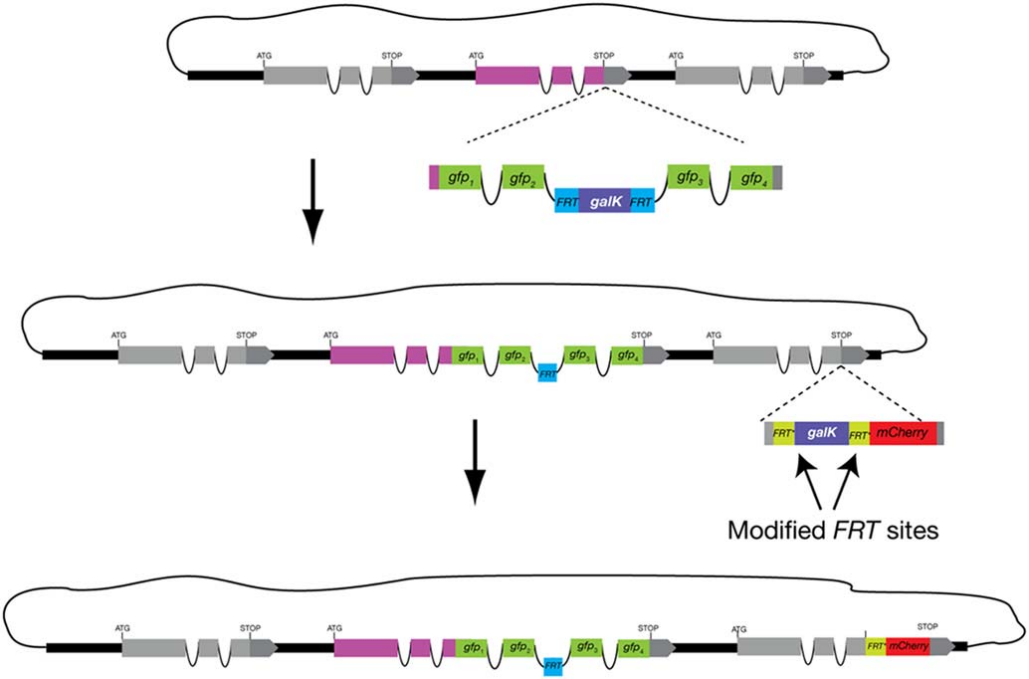
Double recombineering. Two independent manipulations can be carried out on the same fosmid molecule. The two recombinations are done sequentially, each one in the same way as previously described. Note that the second cassette cannot include *FRT* sites as an *FRT* site remains after the first recombination and Flp can potentially recombine any two of the three inserted *FRT* sites. To circumvent this we have introduced an FRT* variant that is still recognized by Flp but does not cross react with the original *FRT* sequence.

Another set of cassettes we have designed allows for double labeling of a single fosmid molecule (Fig. 4). This is of particular use in cases where there are two genes of interest in the same fosmid and labeling with two different colors is desired - such as, for example a pair of paralogous genes. Another potential use is for labeling different splice variants of the same gene. In our strategy the two recombination or labeling reactions are done sequentially. However, one must be careful not to introduce in the second reaction sequences at the end of the linear DNA cassette that are homologous to those inserted in the first one as otherwise the λ Red recombinase would perform unwanted recombination between the two. Homology between interior segments of both cassettes, or between any of the cassettes and the fosmid should not present a problem as the λ Red recombinase has a marked preference for free DNA ends. The remaining *FRT* “scar” from the first recombination reaction does, however, pose a problem for a second recombineering event using Flp recombinase for excision of the selection marker - as recombination could occur between any two of the three inserted *FRT* sites. To avoid this problem, we have modified our cassettes to contain a variant sequence of the *FRT* sites, which we will refer to as FRT* and is still recognized by Flp recombinase but does not recombine with the original *FRT* sequence [17]. Thus, to generate a double recombineered fosmid one could, for example, first insert a GFP tag with the original *FRT-galK-FRT* module and, after flipping out the galK marker, insert a mCherry tag with the modified FRT*-galK-FRT* module (Fig. 4). The end product will be a fosmid with two fluorescent tags and two different 34 bp scars. If one wishes to recombineer a third reporter into a fosmid, one could engineer another variant FRT site into any of our vector cassettes [18].

### Experimental Protocol

Key reagents required for the protocol are listed in Table 2 and a trouble-shooting guide is shown in Table 3.

**Table 2:** Reagents.

***SW105*** ** bacterial strain**	http://recombineering.ncifcrf.gov/
***C.elegans Fosmid library***	http://www.geneservice.co.uk/products/clones/Celegans_Fos.jsp
***LB chloramphenicol (1 liter)***	10 g NaCl; 5 g Yeast Extract; 10 g Bacto-Tryptone; adjust volume to 1 L with H_2_O and autoclave. After cooling to ∼55°C, add 250 µl 50 mg/mL chloramphenicol.
***LB agar plates + chloramphenicol (1 liter)***	10 g NaCl; 5 g Yeast Extract; 10 g Bacto-Tryptone; 15 g Agar; adjust volume to 1 L with H_2_O and autoclave. After cooling to ∼55°C, add 250 µl 50 mg/mL chloramphenicol.
***M9 medium (1 liter)***	6 g Na_2_HPO_4_; 3 g KH_2_PO_4_; 1 g NH_4_Cl; 0.5 g NaCl; adjust volume to 1 L with H_2_O and autoclave.
***Galactose minimal agar plates (1 liter)***	Autoclave 15 g agar in 780 mL H_2_O. After cooling down to ∼55°C add 200 mL 5× M63 medium (10 g (NH_4_)_2_SO_4_; 68 g KH_2_PO_4_; 2.5 mg FeSO_4_·7H_2_O; adjust to pH 7 with KOH; adjust volume to 1 L with H_2_O and autoclave); 1 mL 1 M MgSO_4_·7H_2_O (sterile filtered); 10 ml 20% galactose (autoclaved); 5 mL 0.2 mg/mL d-biotin (sterile filtered); 4.5 mL 10 mg/mL L-leucine (heat up to ∼50°C to dissolve, let cool down- sterile filtered); and 250 µl 50 mg/mL chloramphenicol (in EtOH).
***Deoxy-galactose (DOG) minimal agar plates (1 liter)***	Autoclave 15 g agar in 780 mL H_2_O. After cooling down to ∼55°C add 200 mL 5× M63 medium; 1 mL 1 M MgSO_4_·7H_2_O; 10 ml 20% 2-deoxy-galactose (in H_2_O; autoclaved); 10 ml 20% glycerol (autoclaved); 5 mL 0.2 mg/mL d-biotin; 4.5 mL 10 mg/mL L-leucine; and 250 µl 50 mg/mL chloramphenicol
***MacConkey indicator plates***	Prepare MacConkey agar adding galactose according to manufacturer's instructions (BD Difco, #281810). After autoclaving and cooling to ∼55°C, add 250 µl 50 mg/mL chloramphenicol per liter.
***Arabinose solution***	10% L-arabinose in H_2_O, sterile filtered.

**Table 3:** Troubleshooting.

Problem	Possible solutions
No PCR product with long primers	Vary annealing temperature in a gradient PCR
	Add PCR enhancer solutions such as betaine or DMSO
	Order two sets of shorter primers and add the 50 bp of homology in two sequential reactions
No colonies on *gal* plates after electroporation of the cassette	Confirm quality of competent cells and plates by electroporating control fosmid pBALUxx (contains Chl resistance and the *galK* marker)
	Electroporate more PCR product (up to 300 ng)
	Extend the homology arms of the cassette with an additional pair of primers (to 80–100 bp)
	Titrate heat-shock/induction time (no more than 25–30 min)
	Sequence desired recombineering site in the fosmid to confirm the annotated sequence
Incomplete *galK* excision (evidenced by mixed PCR products)	Make fresh arabinose solution and induce for longer time
	Streak out arabinose-induced bacteria on DOG plates to obtain single colonies that do not contain the *galK* marker
No transgenic lines	Decrease fosmid concentration on array as overexpression might be toxic

#### Choice and isolation of fosmid clones

Fosmid clones that comprise the genomic region of interest can be identified using the Wormbase database at http://www.wormbase.org. The Gbrowse display window should be set to show the genomic region with the gene of interest with underlying fosmid clones as black bars with “WRM…” clone identities. The *C. elegans* fosmid library can be obtained from Geneservice (http://www.geneservice.co.uk/products/clones/Celegans_Fos.jsp). The fosmids are supplied in strain EPI300 (Epicentre) and can be isolated using standard MIDI prep kits.

#### Preparation of electro-competent SW105 bacteria

Strain SW105 can be obtained from Biological Resource Branch at NCI (http://recombineering.ncifcrf.gov/). Inoculate 10 mL LB with a single SW105 colony from a LB agar plate and incubate overnight at 32°C. Note: It is important to keep the temperature at 32°C to avoid activation of the heat inducible λ Red recombinase. Inoculate 100 mL LB in a sterile 1 L flask with 1 mL of the 10 mL starter culture. Grow culture until OD_600_ = 0.4–0.6 and put on ice for 15–20 min. Harvest bacteria in pre-cooled tumblers for 15 min at 4°C with 3000×g. Wash cells with 50 mL ice cold sterile ddH_2_O. Pellet bacteria at 4°C in pre-cooled tumblers for 10–15 min at 3000×g. Wash cells with 50 mL ice cold sterile 12.5% glycerol. Pellet cells in pre-cooled tumblers at 4°C, for 10–15 min at 4000×g. Repeat wash with cold sterile 12.5% glycerol and pellet again. Remove supernatant except for ∼1 mL and resuspend cells. Shock freeze 100 µL aliquots with liquid nitrogen and store at −80°C for up to 12 weeks.

#### Electroporation of fosmids into SW105

For fosmid electroporation into the electro-competent SW105 bacteria chill cuvettes on ice prior to use. Use 50 ng of fosmid DNA and mix with a 100 µL aliquots of SW105. Transfer to pre-chilled electroporation cuvette (0.2 cm electrode gap Gene Pulser, Bio-Rad) and incubate 2 min on ice. Set electroporator (BIO-RAD *E. coli* pulser) to 2.5 KV and activate time constant mode (time constant should be between 4.8–5.4). After electroporation add 900 µL LB immediately and recover cells for 1 h at 32°C with gentle shaking. Plate 1∶10 and 1∶100 dilutions on LB-agar with 12.5 µg/ml chloramphenicol (LB+Chl). Incubate overnight at 32°C.

#### Induction of λ Red recombinase and preparation of electro-competent SW105+fosmid

Inoculate 10 mL LB+Chl with a single colony from the LB-Chl agar plate of SW105 containing the fosmid and incubate overnight at 32°C. Important: keep temperature at 32°C! Inoculate 100 mL LB-Chl in a sterile 1 L flask with 1 mL of the 10 mL starter culture. Grow culture until OD_600_ = 0.4–0.6 and in the meantime set a water bath shaker that fits flasks at 42°C. Incubate the flasks at 42°C for 20 minutes with gentle shaking and transfer flasks to ice for 15–20 min. Harvest bacteria in pre-cooled tumblers for 15 min at 4°C with 3000×g. Wash cells with 50 mL ice cold sterile ddH_2_O. Pellet bacteria at 4°C in pre-cooled tumblers for 10 −15 min at 3000×g. Wash cells with 50 mL ice cold sterile 12.5% glycerol. Pellet cells in pre-cooled tumblers at 4°C, for 10–15 min at 4000×g. Repeat wash with cold sterile 12.5% glycerol and pellet again. Remove supernatant except for ∼1 mL and resuspend cells. Use 100 µL aliquots for electroporation with PCR products that contain the recombineering cassette or shock freeze with liquid Nitrogen to store at −80°C for up to 4 weeks.

#### Primer design and amplification of the recombineering cassette

Choose a suitable cassette from the ones shown in Table 1 for the desired type of labeling (or design your own *FRT-galK-FRT* containing cassette). Cassettes are provided as inserts in the TOPO pCR2.1, pCRII or pCR-XL vectors (Invitrogen) and called “pBALU” (Fig. 3 and Table 1; see Supplement Text S1 for complete sequence of all pBALU vectors). Table 1 provides the 5′ and 3′ end sequences of each cassette such that primers for their amplification can be easily designed. Each primer should carry in addition, at its 5′ end, ∼50 bp of sequence homologous to each side of the site of insertion. Due to the length of the primers they should be purified by polyacrylamide gel electrophoresis to avoid truncated synthesis products. Note that the sequences of the sense strand for the 3′ end are provided such that the flanking region can be added and then the reverse complement of the entire sequence easily obtained and ordered as the 3′ primer. The amplification reaction should be performed with a high-fidelity polymerase such as Pfu Turbo (Stratagene). Because of the length of the primers it is not rare to have to sample a broad range of annealing temperatures before successful amplification is achieved. In addition, it may be necessary to add PCR enhancers (such as 1M betaine or 5% DMSO) or try other polymerases - we have had good results with the Expand Long Template PCR System (Roche). If all these fail, the addition of homology can be done in two consecutive PCR reactions, each one using primers with shorter overhangs. The amplified cassette should be gel purified to remove any template from the PCR reaction and if necessary concentrated for electroporation. Each cassette, except *rla-1* intercistronic-sequence-containing cassette, can also be isolated by EcoRI digestion. The gel-purified, cassette-containing fragment can serve as a template for many PCR reactions. The kanamycin cassette for fosmid extension needs to be generated as a PCR fusion product [6] with the genomic DNA piece that will be added to the fosmid DNA.

#### Electroporation of PCR product into λ Red activated SW105+fosmid

The PCR products that contain the recombineering cassette are electroporated into the electro-competent λ Red-activated, fosmid-containing SW105 bacteria using chilled cuvettes on ice. Use 200 ng of gel purified PCR DNA (in no more than ∼5 µL) and mix with a 100 µL aliquots of the λ Red activated SW105+fosmid. As a negative control, include a sample of mock-electroporated bacteria (i.e. an aliquot of the same heat shocked SW105 but without PCR product). Transfer to the pre-chilled cuvette and incubate 2 min on ice. Set electroporator (BIO-RAD *E. coli* pulser) to 2.5 KV and activate constant time mode. After electroporation add 900 µL LB immediately. Transfer the cell suspension into a 15 mL screw lid tube, wash the cuvette with another 1 mL LB and pool, then incubate for 3 h at room temperature with gentle shaking. Pellet the recovered bacteria and wash once with sterile water to remove the remaining LB. Plate everything on galactose minimal plates containing chloramphenicol or kanamycin if pBALU-ext was used for extension. Incubate chloramphenicol plates for ∼60 h at 32°C or ∼36 h at 32°C for kanamycin.

#### Identification of colonies carrying the recombineered fosmid

Typically, no colonies will be present in the plate from the mock-electroporated bacteria and a few (2–20) will grow in the experimental plates. To confirm that these are indeed *galK*+ and to make sure that single colonies are isolated, streak out 4–6 colonies on a MacConkey indicator plate with galactose and grow over night at 32°C. Bacteria with galactokinase activity will change the pH of the medium and turn red. Pick a single, red colony and grow in 5 mL of LB+Chl to OD600 ∼0.4–0.6 at 32°C (or grow a saturated culture O.N. and then dilute and re-grow to OD600 ∼0.4–0.6 the next day). Split the culture in two and add, to one half, 1/100 volume of 10% arabinose solution (induced sample) and nothing to the rest (un-induced sample). Continue to incubate all cultures at 32°C for 2 h. Harvest both induced and un-induced samples by centrifugation and extract the fosmid by the alkaline lysis method followed by isopropanol precipitation. Typically, we use solutions 1, 2 and 3 from any miniprep kit and rather than loading the lysate on the provided columns we precipitate 800 µL with 0.7 volume of 2-propanol. Resuspend the fosmid-containing pellet in 50–100 µL ddH_2_O and use 1 µL as template for a PCR reaction with primers flanking the site of insertion. Confirm correct insertion of the whole cassette and excision of the *FgF* module by PCR with primers at ∼150–250 bp on each side of the recombination sites. It is helpful to include a reaction with the original fosmid to see the size of an unmodified product. The cassette insertion should cause a size increase of ∼2 kb and excision of the *FgF* a decrease by ∼1.3 Kb.

Fosmids that generate the expected banding pattern in the test PCR are electroporated back into strain EPI300 for proper maintenance. Single EPI300 colonies are grown and used to inoculate ∼100 mL cultures for MIDI-scale DNA extraction. Finally, the recombineered fosmids are sequenced with primers flanking the insertion locus. For further integrity check of the recombineered fosmid diagnostic digestions of the recombineered fosmid and the starting fosmid are recommended. For the kanamycin based extension by recombineering using pBALU-ext the procedure is shorter. The colonies from the kanamycin plate are confirmed by PCR with flanking primers and sequencing for successful extension as described before. In general the SW105 containing the extended fosmid can be used for further *galK* based recombineering.

#### Generating transgenic lines

We typically inject fosmids as mixes with digested bacterial or worm genomic DNA for formation of complex arrays. A unique restriction site in the backbone of the fosmid must be identified for digestion. The digested fosmid concentration in the injection mix is 1–10 ng/uL. The genomic DNA used as carrier is digested with PvuII and added at 150 ng/uL. As injection marker, we typically use *rol-6* added as 2 ng/uL of digested pRF4 [19]. While these complex arrays yield fewer transgenic F1s (about 50% of the yield with undigested, conventional plasmids, injected at higher reporter concentration), the percentage that throw stable transgenic progeny is considerably higher (variable but can reach up to 75%).

For generation of transgenic animals by ballistic transformation [20] it is recommended to use the *C. briggsae* ortholog of *unc-119* since the rescuing genomic fragment is smaller than the *C. elegans unc-119* [10]. We suggest recombining this ∼3.2 kb fragment into the fosmid backbone pCC1FOS (Epicentre) to stably transform animals with the *unc-119* and the recombineered fosmid. For this the *C. briggsae unc-119* could be cloned in conjunction with a selection gene as a plasmid containing flanking homologies to the pCC1FOS backbone and a single restriction site between the homology arms that linearizes the construct. Such a construct would be a universal ready-to-use recombineering cassette to introduce *unc-119* into all fosmids by simply another single step of λ Red-mediated recombination in SW105. We have not further pursued this strategy as in our hands ballistic transformation is more time- and effort- consuming that conventional injection.

#### Availability

The whole vector set described in this manuscript can be obtained on filter paper upon request from the authors. Bacterial strain SW105 can be obtained from NCI-Frederick (http://recombineering.ncifcrf.gov/).

## Results and Discussion

### Example Applications

**Figure 5:**
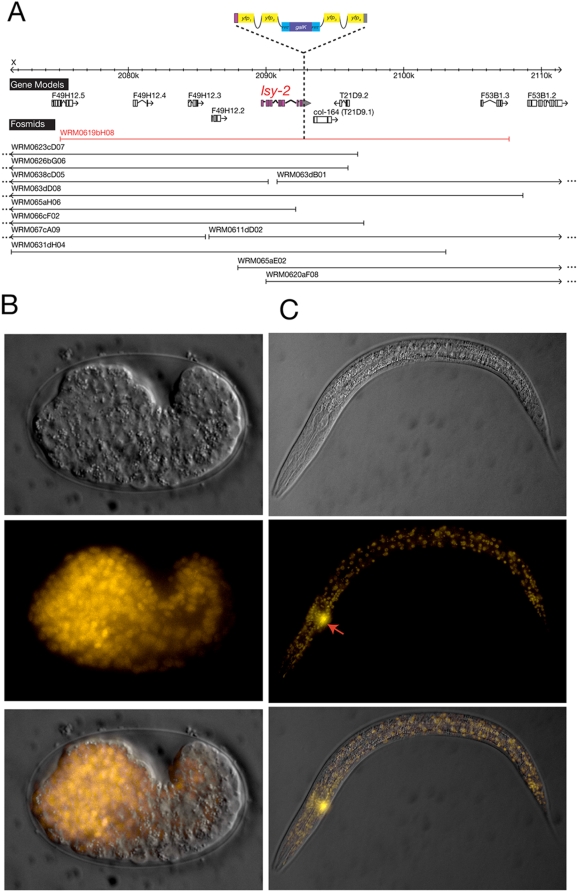
*lsy-2* reporter generated by fosmid recombineering. A. *lsy-2* locus. Complete fosmid coverage of the *lsy-2* locus is shown and the fosmid that was chosen for recombineering is highlighted in red. The pBALU 2 cassette was used to create a C-terminal fusion protein of LSY-2 and YFP. B. Embryonic expression. The recombineered fosmid was injected into *lsy-2* mutant strain *lsy-2(ot90)* mutant containing the ASER specific marker *gcy-5prom::gfp*. The comma stage embryo displays broad expression of *lsy-2::yfp*. The reporter *gcy-5prom::gfp* is not detectable since its earliest expression is at the later 3fold stage. C. Larval expression. The reporter is broadly expressed in larval stages. The arrow indicates *gcy-5::gfp* expression (easily visible through the YFP filter used to visualize LSY-2:YFP), whose loss in *lsy-2(ot90)* animals is completely rescued.

All cassettes, including one with modified FRT* sequences, were tested at least in one color version (Table 1; Supplement Table S1). We describe here some selected, representative results obtained with individual cassettes. We recombineered *gfp*, *cfp* and *yfp* with a hidden, intronic scar into the *lsy-2* locus, which encodes a ubiquitously expressed Zn-finger transcription factor [21]. Each recombineered fosmid rescues the *lsy-2* mutant phenotype (Table 4) and results in broad expression in all tissue types (Fig. 5).

**Table 4:** Rescue of mutant phenotypes with recombineered fosmids.

fosmid recombineered gene *	genetic background	# of lines	complete rescue^1^	partial rescue^2^	no rescue^3^
*che-1::mChOpti (WRM066bC03)*	*che-1(ot94)*	9	7	1	1
*lsy-2::yfp (WRM0619bH08)*	*lsy-2(ot90)*	3	2	0	1

**Figure 6:**
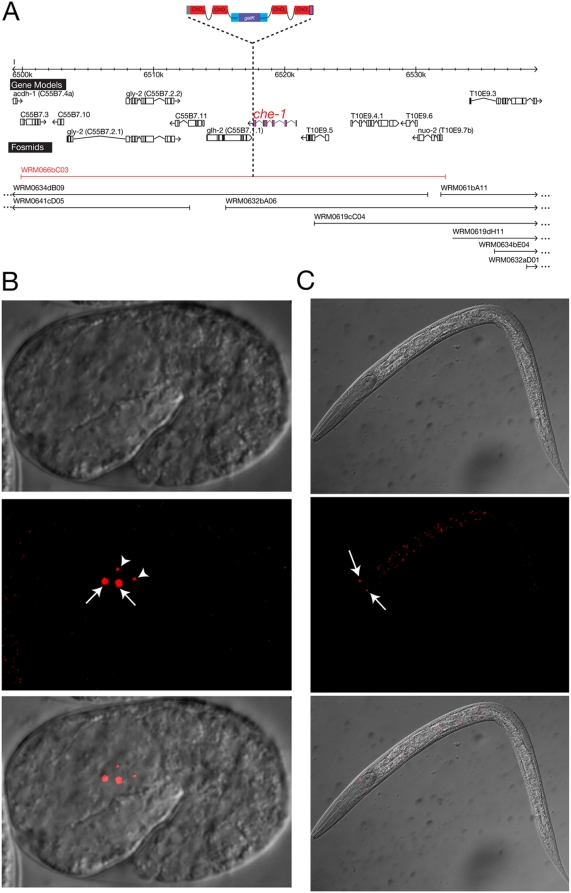
*che-1* reporter generated by fosmid recombineering. A. *che-1* locus. Complete fosmid coverage of the *che-1* locus is shown and the fosmid that was chosen for recombineering is highlighted in red. The pBALU 4 cassette was used to create a C-terminal fusion protein of CHE-1 and mChOpti. B. Embryonic expression. The recombineered fosmid was injected into *che-1(ot94)* mutant animals. The comma stage embryo displays expression of *che-1::mChO* in ASEL and ASER (white arrows) and, briefly, in their sister cells as they undergo apoptosis (white arrowheads). C. Larval expression. *che-1* expression in maintained in the ASEL/R neurons during larval and adult stages.

**Figure 7:**
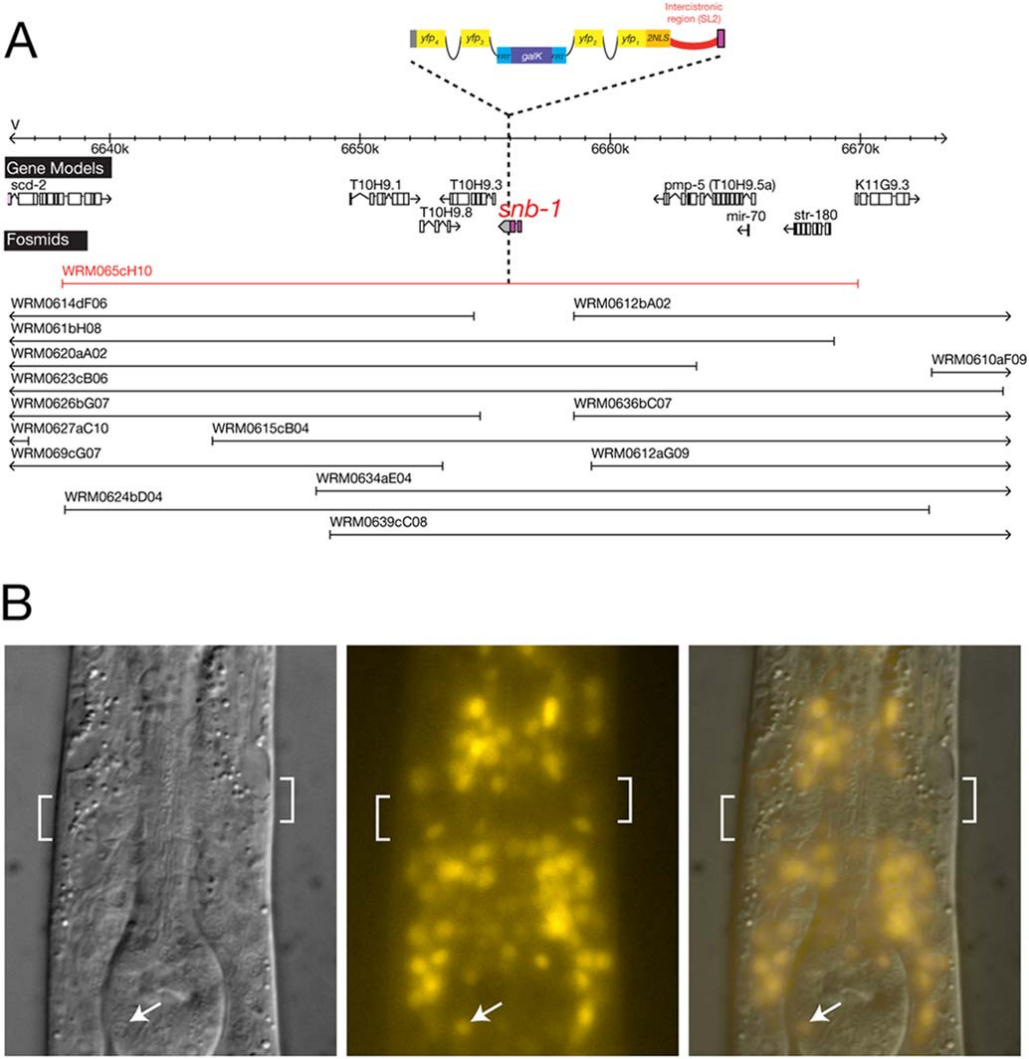
*snb-1* reporter generated by bicistronic fosmid recombineering. A. *snb-1* locus. Complete fosmid coverage of the *snb-1* locus is shown and the fosmid that was chosen for recombineering is highlighted in red. The pBALU 10 cassette was used to create a bicistronic locus to express YFP under the control of *snb-1* promoter and 3′ UTR while not modifying SNB-1 protein. B. Larval expression. The SL2 engineered bicistronic *snb-1* reporter results in *yfp* expression under the control of *snb-1* regulatory elements with the YFP protein being directed to the nucleus of the cells that express *snb-1* in the head region of the L2 larval stage. *snb-1* expression reported by nuclear YFP can be easily assigned to a broad range of distinct neurons in the head. Anterior is to the top. The white brackets indicate the position of the major head neuropil, the nerve ring. The white arrow indicates an isolated neuronal nucleus in the pharynx, identifiable through its speckled nuclear morphology and expressing YFP.

* See Figure 6 and 7 for constructs. *che-1* rescue was assessed by injecting the recombineered fosmid into mutants *ot94; ntIs1 (gcy5prom::gfp)* that display a 100% penetrant total loss of *gfp* expression. Rescued animals restore *gfp* expression in ASER. *lsy-2* rescue was assessed by injecting recombineered fosmids into *ot90; ntIs1 (gcy5prom::gfp)* that display 100% penetrant ectopic *gfp* expression in ASEL. Rescued animals show *gfp* expression only in ASER. For each line at least 20 L4 worms were scored.

1 100% of animals show rescue.

2 >50% of animals show rescue.

3 No rescue and no reporter expression.

Figure 6 describes a reporter construct in which we recombineered mChOpti – a *C. elegans* optimized version of mCherry [13] - with a hidden, intronic scar into the *che-1* locus, which encodes a Zn-finger transcription factor required for the development of the ASE chemosensory neurons [22], [23]. Previous reporter gene analysis with a <10 Kbp reporter construct indicated expression of the gene in ASE, as expected, but also in several other neuron types [22]. Expression in these other neuron types was, however, not stable and the reporter construct also failed to rescue the mutant phenotype of *che-1* [22]. In contrast, our fosmid-recombineered reporter rescues the *che-1* mutant phenotype (Table 4) and reveals expression exclusively and robustly in the ASE neurons, which precisely matches the *che-1* mutant phenotype (Fig. 6). This example therefore illustrates that the inclusion of more genomic regulatory information in the context of fosmid-based reporters produces more reliable and robust expression patterns.

The use of the bicistronic-reporter strategy to monitor nuclear reporter gene expression after fusing a reporter to a non-nuclear protein of interest, can be illustrated with the *snb-1* locus. *snb-1* codes for synaptobrevin, a transmembrane protein that by immunohistochemistry has been shown to localize to synaptic vesicles [24]. As cell identification in *C. elegans* relies on the nuclear position and/or characteristic overall shapes of a cell, such synaptic localization would make it difficult if not impossible to identify the cell types in which the gene is expressed. With the bicistronic approach, we observe expression of the *snb-1* locus in the nucleus of many if not all cells in the nervous system, as expected (Fig. 7).

#### Other Applications and Cassettes

**Figure 8:**
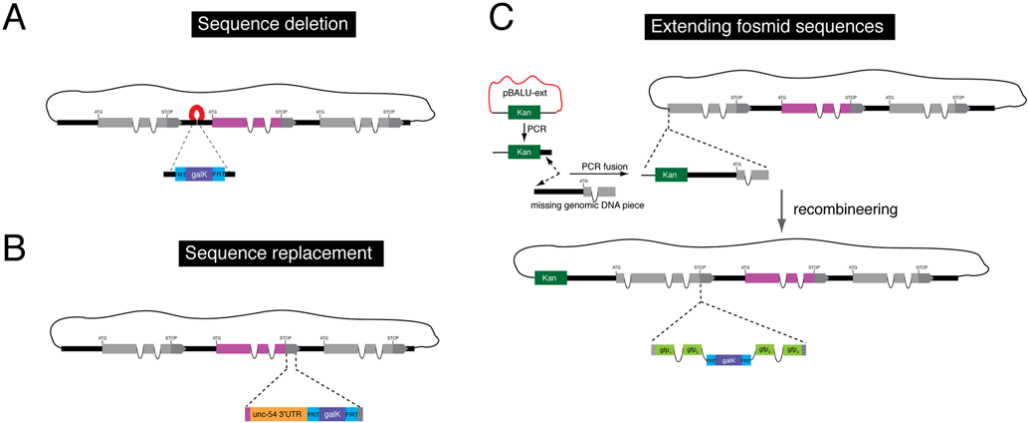
Additional applications of fosmid recombineering. A. *Sequence deletion*. λ Red-mediated recombineering can be used to first replace the sequence to be deleted with the *FRT-galK-FRT* cassette and further Flp-mediated recombination to excise the selectable marker. Note that in this case a 34 bp *FRT* scar will remain in place of the deleted sequence. B. *Sequence replacement*. A similar strategy can be used for sequence replacement. The sequence to be added should be included in the recombineering cassette and co-introduced in the fosmid with the *FRT-galK-FRT* module. As an example we show the replacement of the endogenous 3′ UTR by the generally permissive *C. elegans unc-54* 3′ UTR. This cassette is provided in pBALU21 and can be used to test whether the expression of a given gene is regulated by sequences in its 3′ UTR. C. *Fosmid extension*. Recombineering can be used to extend the genomic insert in a fosmid. The pBALU-ext vector is used to amplify the kanamycin (*Kan*) resistance gene flanked by sequences homologous to the fosmid backbone pCC1FOS and the genomic extension fragment. The genomic fragment is fused by PCR to this cassette generating a *Kan* cassette flanked by the homology to one end of pCC1FOS and the genomic extension fragment. A single λ Red-mediated recombineering step with subsequent *Kan* selection accomplishes the extension with *Kan* gene becoming a permanent part of the fosmid backbone. Note that any of these manipulations can be done in conjunction with additional fluorescent protein or epitope tagging using our double recombineering cassettes with modified *FRT* sites.

There are many other potential variations of this system. Rather than adding a fluorescent reporter, one can recombineer at the 3′end of gene X other heterologous protein-coding regions, such as an ICE-type protease [25] or dominant version of *mec-4* [26] in order to kill exactly those cells in which gene X is normally expressed in (in this particular cases, one would want to separate ICE or *mec-4(d)* with an SL2 sequence from gene X). By recombineering split *gfp* into two different genes, A and B, on two different fosmids, one can test for protein interaction between A and B (“Bimolecular fluorescence complementation”; BiFC)[27] or interactions between cells (“GFP reconstitution across synaptic partners”; GRASP)[28]. Other heterologous sequences, such as a temperature-sensitive *mec-2* intron, that allows for temporally controlled gene expression [29], can also be easily recombineered into a gene of interest. Recombineering can be used to delete sequences, such as for example putative *cis*-regulatory sequences (Fig. 8A), or to delete coding parts of a gene for structure/function analysis. By introducing novel sequences into the cassette, one can also swap sequences present in a fosmid. For example, we have generated a cassette that allows for swapping the 3′UTR of a gene with that of another, canonical 3′UTR (*unc-54*) so as to examine potential regulatory roles of a 3′UTR (Fig. 8B).

As these manipulations usually occur within the context of an already modified (i.e. reporter-tagged) fosmid, it is important to design the experiment such that different FRT sites are used for the first and second manipulation. We have tested this system to replace the 3′UTR of the *cog-1* gene (Supplement Fig.S1), which is normally regulated by its 3′UTR [30].

Recombineering can also be used to extend the genomic regions contained within fosmids (Fig. 8C). The pBALU-ext vector contains a cassette that confers kanamycin (*Kan*) resistance flanked by sequences homologous to the fosmid backbone pCC1FOS. The missing genomic fragment can be PCR-fused [6] on either side of the *Kan* marker in this cassette to generate a fragment that comprises the *Ka*n gene flanked by the homology to the corresponding end of pCC1FOS on one side and the extended genomic fragment on the other (Fig. 8C). A single λ Red mediated recombineering step and subsequent selection for *Kan* resistance accomplishes the extension since the *Kan* gene can be maintained as a permanent part of the backbone. We have tested this vector to extend a fosmid that contains an incomplete *gcy-22* locus (Supplement Fig.S2).

### Conclusion

We have described here a recombineering pipeline that enables the rapid engineering of reporter gene constructs. Our toolkit offers recombineering cassettes for a variety of tagging options that do not affect the functionality of the engineered gene. Our procedure is robust, time- and cost-efficient and easy to adopt as a standard technique for gene expression analysis in *C. elegans*. In addition, the overall approach can be easily adapted for a number of other applications.

## Supporting Information

Text S1 Sequences of all pBALU cassettes
(0.10 MB DOC)

Table S1 Comparison of our protocol to that of Dolphin and Hope
(0.05 MB DOC)

Figure S1 Example of sequence replacement by fosmid recombineering. The goal was to replace the miRNA-regulated 3′UTR from the cog-1 gene with that of 3′UTR from the unc-54 gene. Fosmid WRM067cF11 containing a yfp insertion in the cog-1 gene was used as the substrate for 3′ UTR replacement. pBALU 21 was used as template for amplification of the unc-54 3′ UTR fused to the FgF module flanked by homology to yfp on the 5′ side and to the gene downstream of cog-1 on the 3′ side. Recombination was performed as explained in the main text and resulted in the substitution of the full intergenic region between cog-1 and the downstream gene for the unc-54 3′ UTR and an FRT site. The agarose gel shows the products of PCR amplification with primers A and B using as template the starting fosmid (3), the fosmid with insertion of the full cassette (1) and the fosmid with the 3′ UTR replacement after excision of the FRT-galK module by Flp recombinase (2). The size of all PCR products are as expected and the final product (2) was also confirmed by sequencing.
(0.55 MB TIF)

Figure S2 Example of extending a fosmid sequence. The goal was to extend a fosmid, WRM065cB03, to make it contain the full gcy-22 locus. This application is necessary in those relatively rare cases in which there is low fosmid coverage of a genomic region. pBALU-ext was used to amplify the kanamycin (Kan) resistance gene flanked by sequences homologous to the fosmid backbone pCC1FOS and to the genomic fragment of the gcy-22 gene that is missing from the fosmid. This gcy-22 fragment has an approx. 100 bp overlap with the incomplete gcy-22 sequence in the fosmid and was fused by PCR (Hobert, 2002) to the Kan cassette. In a single recombineering step, with subsequent Kan selection, the extension of the gcy-22 locus was accomplished with the Kan gene becoming a permanent part of the fosmid backbone. The agarose gel shows the PCR products with primers A and B that anneal to the flanking sequence on the fosmid backbone and the incomplete gcy-22. Lane 1 shows the PCR product derived from the non-extended fosmid (515 nt). Lane 2 shows the PCR product using the same primer pair after extension. The size of the PCR product corresponds with the expected size (3343 bp) after successfully completing gcy-22 with the missing fragment and adding the Kan gene to the backbone.
(0.64 MB TIF)
