# Supplementary material for: A Toolkit and Robust Pipeline for the Generation of Fosmid-Based Reporter Genes in C. elegans
Source: PLoS One. 2009 Mar 4;4(3):e4625. doi: 10.1371/journal.pone.0004625 (PMC2649505; doi:10.1371/journal.pone.0004625)
Supplement: Text S1 — Sequences of all pBALU cassettes (0.10 MB DOC) [file pone.0004625.s001.doc]

**The following pages contain the detailed sequences of the pBALU cassettes.**

Where available the pBALU* vectors containing the same corresponding cassette but with modified *FRT* sites are indicated. The sequence modification is as follows:

*FRT* : 5’ gaagttcctatact**ttctag**agaataggaacttc 3’

*FRT**: 5’gaagttcctatact**atttga**agaataggaacttc 3’

The bold, underlined sequence indicates the core sequence that was modified from *FRT* to obtain *FRT**.

All clones were made by PCR fusion and cloned into TOPO-TA based vectors (pCR2.1, pCRII or pCR-XL). All vectors carry a kanamycin resistance gene and M13F and M13R priming sites for sequencing or amplification of the inserted cassette. In addition, all cassettes are flanked by EcoRI restriction sites which can be used to excise the cassette in all cases EXCEPT those containing the rla-1 intercistronic region, as it contains an EcoRI site as well.

**pBALU1/1*: GFP intron-FgF**

atgagtaaaggagaagaacttttcactggagttgtcccaattcttgttgaattagatggtgatgttaatgggcacaaattttctgtcagtggagagggtgaaggtgatgcaacatacggaaaacttacccttaaatttatttgcactactggaaaactacctgttccatgggtaagtttaaacatatatatactaactaaccctgattatttaaattttcagccaacacttgtcactactttctgttatggtgttcaatgcttctcgagatacccagatcatatgaaacggcatgactttttcaagagtgccatgcccgaaggttatgtacaggaaagaactatatttttcaaagatgacgggaactacaagacacgtaagtttaaacagttcggtacgaagttcctatactttctagagaataggaacttccctgttgacaattaatcatcggcatagtatatcggcatagtataatacgacaaggtgaggaactaaacccaggaggcagatcatgagtctgaaagaaaaaacacaatctctgtttgccaacgcatttggctaccctgccactcacaccattcaggcgcctggccgcgtgaatttgattggtgaacacaccgactacaacgacggtttcgttctgccctgcgcgattgattatcaaaccgtgatcagttgtgcaccacgcgatgaccgtaaagttcgcgtgatggcagccgattatgaaaatcagctcgacgagttttccctcgatgcgcccattgtcgcacatgaaaactatcaatgggctaactacgttcgtggcgtggtgaaacatctgcaactgcgtaacaacagcttcggcggcgtggacatggtgatcagcggcaatgtgccgcagggtgccgggttaagttcttccgcttcactggaagtcgcggtcggaaccgtattgcagcagctttatcatctgccgctggacggcgcacaaatcgcgcttaacggtcaggaagcagaaaaccagtttgtaggctgtaactgcgggatcatggatcagctaatttccgcgctcggcaagaaagatcatgccttgctgatcgattgccgctcactggggaccaaagcagtttccatgcccaaaggtgtggctgtcgtcatcatcaacagtaacttcaaacgtaccctggttggcagcgaatacaacacccgtcgtgaacagtgcgaaaccggtgcgcgtttcttccagcagccagccctgcgtgatgtcaccattgaagagttcaacgctgttgcgcatgaactggacccgatcgtggcaaaacgcgtgcgtcatatactgactgaaaacgcccgcaccgttgaagctgccagcgcgctggagcaaggcgacctgaaacgtatgggcgagttgatggcggagtctcatgcctctatgcgcgatgatttcgaaatcaccgtgccgcaaattgacactctggtagaaatcgtcaaagctgtgattggcgacaaaggtggcgtacgcatgaccggcggcggatttggcggctgtatcgtcgcgctgatcccggaagagctggtgcctgccgtacagcaagctgtcgctgaacaatatgaagcaaaaacaggtattaaagagactttttacgtttgtaaaccatcacaaggagcaggacagtgctgaggatccactagttctagagcggccgcgaagttcctatactttctagagaataggaacttctaactaaccatacatatttaaattttcaggtgctgaagtcaagtttgaaggtgatacccttgttaatagaatcgagttaaaaggtattgattttaaagaagatggaaacattcttggacacaaattggaatacaactataactcacacaatgtatacatcatggcagacaaacaaaagaatggaatcaaagttgtaagtttaaacatgattttactaactaactaatctgatttaaattttcagaacttcaaaattagacacaacattgaagatggaagcgttcaactagcagaccattatcaacaaaatactccaattggcgatggccctgtccttttaccagacaaccattacctgtccacacaatctgccctttcgaaagatcccaacgaaaagagagaccacatggtccttcttgagtttgtaacagctgctgggattacacatggcatggatgaactatacaaatag

AATGGAATCAAAGTTgtaagtttaaacatgattttactaactaactaatctgatttaaattttcagAACTTCAAAATTAGACACAAC

------- GFP

------- FRT sites

------- galK

------- artificial introns

**pBALU2/2*: YFP intron-FgF**

atgagtaaaggagaagaacttttcactggagttgtcccaattcttgttgaattagatggtgatgttaatgggcacaaattttctgtcagtggagagggtgaaggtgatgcaacatacggaaaacttacccttaaatttatttgcactactggaaaactacctgttccatgggtaagtttaaacatatatatactaactaaccctgattatttaaattttcagccaacacttgtcactactttcggttatggtcttcaatgcttcgccagatacccagatcatatgaaacagcatgactttttcaagagtgccatgcccgaaggttatgtacaggaaagaactatatttttcaaagatgacgggaactacaagacacgtaagtttaaacagttcggtacgaagttcctatactttctagagaataggaacttccctgttgacaattaatcatcggcatagtatatcggcatagtataatacgacaaggtgaggaactaaacccaggaggcagatcatgagtctgaaagaaaaaacacaatctctgtttgccaacgcatttggctaccctgccactcacaccattcaggcgcctggccgcgtgaatttgattggtgaacacaccgactacaacgacggtttcgttctgccctgcgcgattgattatcaaaccgtgatcagttgtgcaccacgcgatgaccgtaaagttcgcgtgatggcagccgattatgaaaatcagctcgacgagttttccctcgatgcgcccattgtcgcacatgaaaactatcaatgggctaactacgttcgtggcgtggtgaaacatctgcaactgcgtaacaacagcttcggcggcgtggacatggtgatcagcggcaatgtgccgcagggtgccgggttaagttcttccgcttcactggaagtcgcggtcggaaccgtattgcagcagctttatcatctgccgctggacggcgcacaaatcgcgcttaacggtcaggaagcagaaaaccagtttgtaggctgtaactgcgggatcatggatcagctaatttccgcgctcggcaagaaagatcatgccttgctgatcgattgccgctcactggggaccaaagcagtttccatgcccaaaggtgtggctgtcgtcatcatcaacagtaacttcaaacgtaccctggttggcagcgaatacaacacccgtcgtgaacagtgcgaaaccggtgcgcgtttcttccagcagccagccctgcgtgatgtcaccattgaagagttcaacgctgttgcgcatgaactggacccgatcgtggcaaaacgcgtgcgtcatatactgactgaaaacgcccgcaccgttgaagctgccagcgcgctggagcaaggcgacctgaaacgtatgggcgagttgatggcggagtctcatgcctctatgcgcgatgatttcgaaatcaccgtgccgcaaattgacactctggtagaaatcgtcaaagctgtgattggcgacaaaggtggcgtacgcatgaccggcggcggatttggcggctgtatcgtcgcgctgatcccggaagagctggtgcctgccgtacagcaagctgtcgctgaacaatatgaagcaaaaacaggtattaaagagactttttacgtttgtaaaccatcacaaggagcaggacagtgctgaggatccactagttctagagcggccgcgaagttcctatactttctagagaataggaacttctaactaaccatacatatttaaattttcaggtgctgaagtcaagtttgaaggtgatacccttgttaatagaatcgagttaaaaggtattgattttaaagaagatggaaacattcttggacacaaattggaatacaactataactcacacaatgtatacatcatggcagacaaacaaaagaatggaatcaaagttgtaagtttaaacatgattttactaactaactaatctgatttaaattttcagaacttcaaaattagacacaacattgaagatggaagcgttcaactagcagaccattatcaacaaaatactccaattggcgatggccctgtccttttaccagacaaccattacctgtcctaccaatctgccctttcgaaagatcccaacgaaaagagagaccacatggtccttcttgagtttgtaacagctgctgggattacacatggcatggatgaactatacaaatag

------- YFP

------- FRT sites

------- galK

------- artificial introns

**pBALU3/3*: CFP_intron-FgF**

atgagtaaaggagaagaacttttcactggagttgtcccaattcttgttgaattagatggtgatgttaatgggcacaaattttctgtcagtggagagggtgaaggtgatgcaacatacggaaaacttacccttaaatttatttgcactactggaaaactacctgttccatgggtaagtttaaacatatatatactaactaaccctgattatttaaattttcagccaacacttgtcactactttctcatggggcgttcaatgcttctcgagatacccagatcatatgaaacagcatgactttttcaagagtgccatgcccgaaggttatgtacaggaaagaactatatttttcaaagatgacgggaactacaagacacgtaagtttaaacagttcggtacgaagttcctatactttctagagaataggaacttccctgttgacaattaatcatcggcatagtatatcggcatagtataatacgacaaggtgaggaactaaacccaggaggcagatcatgagtctgaaagaaaaaacacaatctctgtttgccaacgcatttggctaccctgccactcacaccattcaggcgcctggccgcgtgaatttgattggtgaacacaccgactacaacgacggtttcgttctgccctgcgcgattgattatcaaaccgtgatcagttgtgcaccacgcgatgaccgtaaagttcgcgtgatggcagccgattatgaaaatcagctcgacgagttttccctcgatgcgcccattgtcgcacatgaaaactatcaatgggctaactacgttcgtggcgtggtgaaacatctgcaactgcgtaacaacagcttcggcggcgtggacatggtgatcagcggcaatgtgccgcagggtgccgggttaagttcttccgcttcactggaagtcgcggtcggaaccgtattgcagcagctttatcatctgccgctggacggcgcacaaatcgcgcttaacggtcaggaagcagaaaaccagtttgtaggctgtaactgcgggatcatggatcagctaatttccgcgctcggcaagaaagatcatgccttgctgatcgattgccgctcactggggaccaaagcagtttccatgcccaaaggtgtggctgtcgtcatcatcaacagtaacttcaaacgtaccctggttggcagcgaatacaacacccgtcgtgaacagtgcgaaaccggtgcgcgtttcttccagcagccagccctgcgtgatgtcaccattgaagagttcaacgctgttgcgcatgaactggacccgatcgtggcaaaacgcgtgcgtcatatactgactgaaaacgcccgcaccgttgaagctgccagcgcgctggagcaaggcgacctgaaacgtatgggcgagttgatggcggagtctcatgcctctatgcgcgatgatttcgaaatcaccgtgccgcaaattgacactctggtagaaatcgtcaaagctgtgattggcgacaaaggtggcgtacgcatgaccggcggcggatttggcggctgtatcgtcgcgctgatcccggaagagctggtgcctgccgtacagcaagctgtcgctgaacaatatgaagcaaaaacaggtattaaagagactttttacgtttgtaaaccatcacaaggagcaggacagtgctgaggatccactagttctagagcggccgcgaagttcctatactttctagagaataggaacttctaactaaccatacatatttaaattttcaggtgctgaagtcaagtttgaaggtgatacccttgttaatagaatcgagttaaaaggtattgattttaaagaagatggaaacattcttggacacaaattggaatacaactatatttcacacaacgtatacatcactgccgacaaacaaaagaatgggatcaaagcggtaagtttaaacatgattttactaactaactaatctgatttaaattttcagaacttcaaaattagacacaacattgaagatggaagcgttcaactagcagaccattatcaacaaaatactccaattggcgatggccctgtccttttaccagacaaccattacctgtccacacaatctgccctttcgaaagatcccaacgaaaagagagaccacatggtccttcttgagtttgtaacagctgctgggattacacatggcatggatgaactatacaaatag

------- CFP

------- FRT sites

------- galK

------- artificial introns

**pBALU4/4*: mChOpti_intron-FgF**

atggtctcaaagggtgaagaagataacatggcaattattaaagagtttatgcgtttcaaggtgcatatggagggatctgtcaatgggcatgagtttgaaattgaaggtgaaggagaaggccgaccatatgagggaacacaaaccgcaaaactaaaggtaagtttaaacatatatatactaactaaccctgattatttaaattttcaggtaactaaaggcggaccattaccattcgcctgggacatcctctctccacagttcatgtatggaagtaaagcttatgttaaacatccggcagatataccagattatttgaaactttcattcccggagggttttaagtgggaacgcgtaatgaattttgaagacggaggagttgttacagtgacgcaagactcaaggtaagtttaaacagttcggtacgaagttcctatactttctagagaataggaacttccctgttgacaattaatcatcggcatagtatatcggcatagtataatacgacaaggtgaggaactaaacccaggaggcagatcatgagtctgaaagaaaaaacacaatctctgtttgccaacgcatttggctaccctgccactcacaccattcaggcgcctggccgcgtgaatttgattggtgaacacaccgactacaacgacggtttcgttctgccctgcgcgattgattatcaaaccgtgatcagttgtgcaccacgcgatgaccgtaaagttcgcgtgatggcagccgattatgaaaatcagctcgacgagttttccctcgatgcgcccattgtcgcacatgaaaactatcaatgggctaactacgttcgtggcgtggtgaaacatctgcaactgcgtaacaacagcttcggcggcgtggacatggtgatcagcggcaatgtgccgcagggtgccgggttaagttcttccgcttcactggaagtcgcggtcggaaccgtattgcagcagctttatcatctgccgctggacggcgcacaaatcgcgcttaacggtcaggaagcagaaaaccagtttgtaggctgtaactgcgggatcatggatcagctaatttccgcgctcggcaagaaagatcatgccttgctgatcgattgccgctcactggggaccaaagcagtttccatgcccaaaggtgtggctgtcgtcatcatcaacagtaacttcaaacgtaccctggttggcagcgaatacaacacccgtcgtgaacagtgcgaaaccggtgcgcgtttcttccagcagccagccctgcgtgatgtcaccattgaagagttcaacgctgttgcgcatgaactggacccgatcgtggcaaaacgcgtgcgtcatatactgactgaaaacgcccgcaccgttgaagctgccagcgcgctggagcaaggcgacctgaaacgtatgggcgagttgatggcggagtctcatgcctctatgcgcgatgatttcgaaatcaccgtgccgcaaattgacactctggtagaaatcgtcaaagctgtgattggcgacaaaggtggcgtacgcatgaccggcggcggatttggcggctgtatcgtcgcgctgatcccggaagagctggtgcctgccgtacagcaagctgtcgctgaacaatatgaagcaaaaacaggtattaaagagactttttacgtttgtaaaccatcacaaggagcaggacagtgctgaggatccactagttctagagcggccgcgaagttcctatactttctagagaataggaacttctaactaaccatacatatttaaattttcagcctccaagatggagaatttatttataaagtcaaacttcgaggaacgaatttcccctcggatggacctgttatgcagaagaagactatgggatgggaagcttcaagtgaaagaatgtaccctgaagacggtgctcttaagggagagattaaacaacgtcttaaattgaaagatggaggacattacgatgctgaggtaagtttaaacatgattttactaactaactaatctgatttaaattttcaggtgaagacaacttacaaagccaaaaaaccagttcagctgccaggagcgtacaatgttaatattaaactggatatcacctcccacaacgaggattacactatcgttgagcaatatgaaagagctgaagggcggcactcgacaggtggcatggatgaattgtataagtaa

------- mChOpti

------- FRT sites

------- galK

------- artificial introns

**pBALU5/5*: FgF-2xFLAG-Venus**

gggaagttcctatactttctagagaataggaacttccctgttgacaattaatcatcggcatagtatatcggcatagtataatacgacaaggtgaggaactaaacccaggaggcagatcatgagtctgaaagaaaaaacacaatctctgtttgccaacgcatttggctaccctgccactcacaccattcaggcgcctggccgcgtgaatttgattggtgaacacaccgactacaacgacggtttcgttctgccctgcgcgattgattatcaaaccgtgatcagttgtgcaccacgcgatgaccgtaaagttcgcgtgatggcagccgattatgaaaatcagctcgacgagttttccctcgatgcgcccattgtcgcacatgaaaactatcaatgggctaactacgttcgtggcgtggtgaaacatctgcaactgcgtaacaacagcttcggcggcgtggacatggtgatcagcggcaatgtgccgcagggtgccgggttaagttcttccgcttcactggaagtcgcggtcggaaccgtattgcagcagctttatcatctgccgctggacggcgcacaaatcgcgcttaacggtcaggaagcagaaaaccagtttgtaggctgtaactgcgggatcatggatcagctaatttccgcgctcggcaagaaagatcatgccttgctgatcgattgccgctcactggggaccaaagcagtttccatgcccaaaggtgtggctgtcgtcatcatcaacagtaacttcaaacgtaccctggttggcagcgaatacaacacccgtcgtgaacagtgcgaaaccggtgcgcgtttcttccagcagccagccctgcgtgatgtcaccattgaagagttcaacgctgttgcgcatgaactggacccgatcgtggcaaaacgcgtgcgtcatatactgactgaaaacgcccgcaccgttgaagctgccagcgcgctggagcaaggcgacctgaaacgtatgggcgagttgatggcggagtctcatgcctctatgcgcgatgatttcgaaatcaccgtgccgcaaattgacactctggtagaaatcgtcaaagctgtgattggcgacaaaggtggcgtacgcatgaccggcggcggatttggcggctgtatcgtcgcgctgatcccggaagagctggtgcctgccgtacagcaagctgtcgctgaacaatatgaagcaaaaacaggtattaaagagactttttacgtttgtaaaccatcacaaggagcaggacagtgctgaggatccactagttctagagcggccgcgaagttcctatactttctagagaataggaacttcgactacaaggacgacgacgacaagggaggagactataaggatgacgatgacaaatctggaggaggaggatctatggtgagcaagggcgaggagctgttcaccggggtggtgcccatcctggtcgagctggacggcgacgtaaacggccacaagttcagcgtgtccggcgagggcgagggcgatgccacctacggcaagctgaccctgaagctgatctgcaccaccggcaagctgcccgtgccctggcccaccctcgtgaccaccctgggctacggcctgcagtgcttcgcccgctaccccgaccacatgaagcagcacgacttcttcaagtccgccatgcccgaaggctacgtccaggagcgcaccatcttcttcaaggacgacggcaactacaagacccgcgccgaggtgaagttcgagggcgacaccctggtgaaccgcatcgagctgaagggcatcgacttcaaggaggacggcaacatcctggggcacaagctggagtacaactacaacagccacaacgtctatatcaccgccgacaagcagaagaacggcatcaaggccaacttcaagatccgccacaacatcgaggacggcggcgtgcagctcgccgaccactaccagcagaacacccccatcggcgacggccccgtgctgctgcccgacaaccactacctgagctaccagtccgccctgagcaaagaccccaacgagaagcgcgatcacatggtcctgctggagttcgtgaccgccgccgggatcactctcggcatggacgagctgtacaagtaa

------- important for “frame”

------- FRT sites

------- galK

------- 2xFLAG

------- VENUS

**pBALU6: Venus-FgF**

atggtgagcaagggcgaggagctgttcaccggggtggtgcccatcctggtcgagctggacggcgacgtaaacggccacaagttcagcgtgtccggcgagggcgagggcgatgccacctacggcaagctgaccctgaagctgatctgcaccaccggcaagctgcccgtgccctggcccaccctcgtgaccaccctgggctacggcctgcagtgcttcgcccgctaccccgaccacatgaagcagcacgacttcttcaagtccgccatgcccgaaggctacgtccaggagcgcaccatcttcttcaaggacgacggcaactacaagacccgcgccgaggtgaagttcgagggcgacaccctggtgaaccgcatcgagctgaagggcatcgacttcaaggaggacggcaacatcctggggcacaagctggagtacaactacaacagccacaacgtctatatcaccgccgacaagcagaagaacggcatcaaggccaacttcaagatccgccacaacatcgaggacggcggcgtgcagctcgccgaccactaccagcagaacacccccatcggcgacggccccgtgctgctgcccgacaaccactacctgagctaccagtccgccctgagcaaagaccccaacgagaagcgcgatcacatggtcctgctggagttcgtgaccgccgccgggatcactctcggcatggacgagctgtacaaggggaagttcctatactttctagagaataggaacttccctgttgacaattaatcatcggcatagtatatcggcatagtataatacgacaaggtgaggaactaaacccaggaggcagatcatgagtctgaaagaaaaaacacaatctctgtttgccaacgcatttggctaccctgccactcacaccattcaggcgcctggccgcgtgaatttgattggtgaacacaccgactacaacgacggtttcgttctgccctgcgcgattgattatcaaaccgtgatcagttgtgcaccacgcgatgaccgtaaagttcgcgtgatggcagccgattatgaaaatcagctcgacgagttttccctcgatgcgcccattgtcgcacatgaaaactatcaatgggctaactacgttcgtggcgtggtgaaacatctgcaactgcgtaacaacagcttcggcggcgtggacatggtgatcagcggcaatgtgccgcagggtgccgggttaagttcttccgcttcactggaagtcgcggtcggaaccgtattgcagcagctttatcatctgccgctggacggcgcacaaatcgcgcttaacggtcaggaagcagaaaaccagtttgtaggctgtaactgcgggatcatggatcagctaatttccgcgctcggcaagaaagatcatgccttgctgatcgattgccgctcactggggaccaaagcagtttccatgcccaaaggtgtggctgtcgtcatcatcaacagtaacttcaaacgtaccctggttggcagcgaatacaacacccgtcgtgaacagtgcgaaaccggtgcgcgtttcttccagcagccagccctgcgtgatgtcaccattgaagagttcaacgctgttgcgcatgaactggacccgatcgtggcaaaacgcgtgcgtcatatactgactgaaaacgcccgcaccgttgaagctgccagcgcgctggagcaaggcgacctgaaacgtatgggcgagttgatggcggagtctcatgcctctatgcgcgatgatttcgaaatcaccgtgccgcaaattgacactctggtagaaatcgtcaaagctgtgattggcgacaaaggtggcgtacgcatgaccggcggcggatttggcggctgtatcgtcgcgctgatcccggaagagctggtgcctgccgtacagcaagctgtcgctgaacaatatgaagcaaaaacaggtattaaagagactttttacgtttgtaaaccatcacaaggagcaggacagtgctgaggatccactagttctagagcggccgcgaagttcctatactttctagagaataggaacttc

------- VENUS

------- important for “frame”

------- FRT sites

------- galK

**pBALU7/7*: FgF-mCherry**

gggaagttcctatactttctagagaataggaacttccctgttgacaattaatcatcggcatagtatatcggcatagtataatacgacaaggtgaggaactaaacccaggaggcagatcatgagtctgaaagaaaaaacacaatctctgtttgccaacgcatttggctaccctgccactcacaccattcaggcgcctggccgcgtgaatttgattggtgaacacaccgactacaacgacggtttcgttctgccctgcgcgattgattatcaaaccgtgatcagttgtgcaccacgcgatgaccgtaaagttcgcgtgatggcagccgattatgaaaatcagctcgacgagttttccctcgatgcgcccattgtcgcacatgaaaactatcaatgggctaactacgttcgtggcgtggtgaaacatctgcaactgcgtaacaacagcttcggcggcgtggacatggtgatcagcggcaatgtgccgcagggtgccgggttaagttcttccgcttcactggaagtcgcggtcggaaccgtattgcagcagctttatcatctgccgctggacggcgcacaaatcgcgcttaacggtcaggaagcagaaaaccagtttgtaggctgtaactgcgggatcatggatcagctaatttccgcgctcggcaagaaagatcatgccttgctgatcgattgccgctcactggggaccaaagcagtttccatgcccaaaggtgtggctgtcgtcatcatcaacagtaacttcaaacgtaccctggttggcagcgaatacaacacccgtcgtgaacagtgcgaaaccggtgcgcgtttcttccagcagccagccctgcgtgatgtcaccattgaagagttcaacgctgttgcgcatgaactggacccgatcgtggcaaaacgcgtgcgtcatatactgactgaaaacgcccgcaccgttgaagctgccagcgcgctggagcaaggcgacctgaaacgtatgggcgagttgatggcggagtctcatgcctctatgcgcgatgatttcgaaatcaccgtgccgcaaattgacactctggtagaaatcgtcaaagctgtgattggcgacaaaggtggcgtacgcatgaccggcggcggatttggcggctgtatcgtcgcgctgatcccggaagagctggtgcctgccgtacagcaagctgtcgctgaacaatatgaagcaaaaacaggtattaaagagactttttacgtttgtaaaccatcacaaggagcaggacagtgctgaggatccactagttctagagcggccgcgaagttcctatactttctagagaataggaacttcgtgagcaagggcgaggaggataacatggccatcatcaaggagttcatgcgcttcaaggtgcacatggagggctccgtgaacggccacgagttcgagatcgagggcgagggcgagggccgcccctacgagggcacccagaccgccaagctgaaggtgaccaagggtggccccctgcccttcgcctgggacatcctgtcccctcagttcatgtacggctccaaggcctacgtgaagcaccccgccgacatccccgactacttgaagctgtccttccccgagggcttcaagtgggagcgcgtgatgaacttcgaggacggcggcgtggtgaccgtgacccaggactcctccctgcaggacggcgagttcatctacaaggtgaagctgcgcggcaccaacttcccctccgacggccccgtaatgcagaagaagaccatgggctgggaggcctcctccgagcggatgtaccccgaggacggcgccctgaagggcgagatcaagcagaggctgaagctgaaggacggcggccactacgacgctgaggtcaagaccacctacaaggccaagaagcccgtgcagctgcccggcgcctacaacgtcaacatcaagttggacatcacctcccacaacgaggactacaccatcgtggaacagtacgaacgcgccgagggccgccactccaccggcggcatggacgagctgtacaagtaa

------- important for “frame”

------- FRT sites

------- galK

------- mCherry

**pBALU8: mCherry-FgF**

atggtgagcaagggcgaggaggataacatggccatcatcaaggagttcatgcgcttcaaggtgcacatggagggctccgtgaacggccacgagttcgagatcgagggcgagggcgagggccgcccctacgagggcacccagaccgccaagctgaaggtgaccaagggtggccccctgcccttcgcctgggacatcctgtcccctcagttcatgtacggctccaaggcctacgtgaagcaccccgccgacatccccgactacttgaagctgtccttccccgagggcttcaagtgggagcgcgtgatgaacttcgaggacggcggcgtggtgaccgtgacccaggactcctccctgcaggacggcgagttcatctacaaggtgaagctgcgcggcaccaacttcccctccgacggccccgtaatgcagaagaagaccatgggctgggaggcctcctccgagcggatgtaccccgaggacggcgccctgaagggcgagatcaagcagaggctgaagctgaaggacggcggccactacgacgctgaggtcaagaccacctacaaggccaagaagcccgtgcagctgcccggcgcctacaacgtcaacatcaagttggacatcacctcccacaacgaggactacaccatcgtggaacagtacgaacgcgccgagggccgccactccaccggcggcatggacgagctgtacaaggggaagttcctatactttctagagaataggaacttccctgttgacaattaatcatcggcatagtatatcggcatagtataatacgacaaggtgaggaactaaacccaggaggcagatcatgagtctgaaagaaaaaacacaatctctgtttgccaacgcatttggctaccctgccactcacaccattcaggcgcctggccgcgtgaatttgattggtgaacacaccgactacaacgacggtttcgttctgccctgcgcgattgattatcaaaccgtgatcagttgtgcaccacgcgatgaccgtaaagttcgcgtgatggcagccgattatgaaaatcagctcgacgagttttccctcgatgcgcccattgtcgcacatgaaaactatcaatgggctaactacgttcgtggcgtggtgaaacatctgcaactgcgtaacaacagcttcggcggcgtggacatggtgatcagcggcaatgtgccgcagggtgccgggttaagttcttccgcttcactggaagtcgcggtcggaaccgtattgcagcagctttatcatctgccgctggacggcgcacaaatcgcgcttaacggtcaggaagcagaaaaccagtttgtaggctgtaactgcgggatcatggatcagctaatttccgcgctcggcaagaaagatcatgccttgctgatcgattgccgctcactggggaccaaagcagtttccatgcccaaaggtgtggctgtcgtcatcatcaacagtaacttcaaacgtaccctggttggcagcgaatacaacacccgtcgtgaacagtgcgaaaccggtgcgcgtttcttccagcagccagccctgcgtgatgtcaccattgaagagttcaacgctgttgcgcatgaactggacccgatcgtggcaaaacgcgtgcgtcatatactgactgaaaacgcccgcaccgttgaagctgccagcgcgctggagcaaggcgacctgaaacgtatgggcgagttgatggcggagtctcatgcctctatgcgcgatgatttcgaaatcaccgtgccgcaaattgacactctggtagaaatcgtcaaagctgtgattggcgacaaaggtggcgtacgcatgaccggcggcggatttggcggctgtatcgtcgcgctgatcccggaagagctggtgcctgccgtacagcaagctgtcgctgaacaatatgaagcaaaaacaggtattaaagagactttttacgtttgtaaaccatcacaaggagcaggacagtgctgaggatccactagttctagagcggccgcgaagttcctatactttctagagaataggaacttc

------- mCherry

------- important for “frame”

------- FRT sites

------- galK

**pBALU9: gpd-2 intergenic + GFP intron-FgF**

gctgtctcatcctactttcacctagttaactgcttgtcttaaaatctatgcttctctttagtatctaaaattttcctagaagcttacaagtatataaatggtctcttctcaataaaggttgtatatttattcatcttattgaatctgccatttcctcgtttttgcgagtttatataccttccaattttctttctattgtattttcaacttctaattttaattcagggaaactgcttcaacgcatcatgaccgctccaaagaagaaacgcaaagtaccggtagaaaaaatgagtaaaggagaagaacttttcactggagttgtcccaattcttgttgaattagatggtgatgttaatgggcacaaattttctgtcagtggagagggtgaaggtgatgcaacatacggaaaacttacccttaaatttatttgcactactggaaaactacctgttccatgggtaagtttaaacatatatatactaactaaccctgattatttaaattttcagccaacacttgtcactactttctgttatggtgttcaatgcttctcgagatacccagatcatatgaaacggcatgactttttcaagagtgccatgcccgaaggttatgtacaggaaagaactatatttttcaaagatgacgggaactacaagacacgtaagtttaaacagttcggtacgaagttcctatactttctagagaataggaacttccctgttgacaattaatcatcggcatagtatatcggcatagtataatacgacaaggtgaggaactaaacccaggaggcagatcatgagtctgaaagaaaaaacacaatctctgtttgccaacgcatttggctaccctgccactcacaccattcaggcgcctggccgcgtgaatttgattggtgaacacaccgactacaacgacggtttcgttctgccctgcgcgattgattatcaaaccgtgatcagttgtgcaccacgcgatgaccgtaaagttcgcgtgatggcagccgattatgaaaatcagctcgacgagttttccctcgatgcgcccattgtcgcacatgaaaactatcaatgggctaactacgttcgtggcgtggtgaaacatctgcaactgcgtaacaacagcttcggcggcgtggacatggtgatcagcggcaatgtgccgcagggtgccgggttaagttcttccgcttcactggaagtcgcggtcggaaccgtattgcagcagctttatcatctgccgctggacggcgcacaaatcgcgcttaacggtcaggaagcagaaaaccagtttgtaggctgtaactgcgggatcatggatcagctaatttccgcgctcggcaagaaagatcatgccttgctgatcgattgccgctcactggggaccaaagcagtttccatgcccaaaggtgtggctgtcgtcatcatcaacagtaacttcaaacgtaccctggttggcagcgaatacaacacccgtcgtgaacagtgcgaaaccggtgcgcgtttcttccagcagccagccctgcgtgatgtcaccattgaagagttcaacgctgttgcgcatgaactggacccgatcgtggcaaaacgcgtgcgtcatatactgactgaaaacgcccgcaccgttgaagctgccagcgcgctggagcaaggcgacctgaaacgtatgggcgagttgatggcggagtctcatgcctctatgcgcgatgatttcgaaatcaccgtgccgcaaattgacactctggtagaaatcgtcaaagctgtgattggcgacaaaggtggcgtacgcatgaccggcggcggatttggcggctgtatcgtcgcgctgatcccggaagagctggtgcctgccgtacagcaagctgtcgctgaacaatatgaagcaaaaacaggtattaaagagactttttacgtttgtaaaccatcacaaggagcaggacagtgctgaggatccactagttctagagcggccgcgaagttcctatactttctagagaataggaacttctaactaaccatacatatttaaattttcaggtgctgaagtcaagtttgaaggtgatacccttgttaatagaatcgagttaaaaggtattgattttaaagaagatggaaacattcttggacacaaattggaatacaactataactcacacaatgtatacatcatggcagacaaacaaaagaatggaatcaaagttgtaagtttaaacatgattttactaactaactaatctgatttaaattttcagaacttcaaaattagacacaacattgaagatggaagcgttcaactagcagaccattatcaacaaaatactccaattggcgatggccctgtccttttaccagacaaccattacctgtccacacaatctgccctttcgaaagatcccaacgaaaagagagaccacatggtccttcttgagtttgtaacagctgctgggattacacatggcatggatgaactatacaaatag

------- gpd-2 intergenic region

------- 1xNLS

------- GFP

------- FRT sites

------- galK

------- artificial introns

**pBALU10: gpd-2 intergenic + YFP intron-FgF**

gctgtctcatcctactttcacctagttaactgcttgtcttaaaatctatgcttctctttagtatctaaaattttcctagaagcttacaagtatataaatggtctcttctcaataaaggttgtatatttattcatcttattgaatctgccatttcctcgtttttgcgagtttatataccttccaattttctttctattgtattttcaacttctaattttaattcagggaaactgcttcaacgcatcatgaccgctccaaagaagaaacgcaaagtaccggtagaaaaaatgagtaaaggagaagaacttttcactggagttgtcccaattcttgttgaattagatggtgatgttaatgggcacaaattttctgtcagtggagagggtgaaggtgatgcaacatacggaaaacttacccttaaatttatttgcactactggaaaactacctgttccatgggtaagtttaaacatatatatactaactaaccctgattatttaaattttcagccaacacttgtcactactttcggttatggtcttcaatgcttcgccagatacccagatcatatgaaacagcatgactttttcaagagtgccatgcccgaaggttatgtacaggaaagaactatatttttcaaagatgacgggaactacaagacacgtaagtttaaacagttcggtacgaagttcctatactttctagagaataggaacttccctgttgacaattaatcatcggcatagtatatcggcatagtataatacgacaaggtgaggaactaaacccaggaggcagatcatgagtctgaaagaaaaaacacaatctctgtttgccaacgcatttggctaccctgccactcacaccattcaggcgcctggccgcgtgaatttgattggtgaacacaccgactacaacgacggtttcgttctgccctgcgcgattgattatcaaaccgtgatcagttgtgcaccacgcgatgaccgtaaagttcgcgtgatggcagccgattatgaaaatcagctcgacgagttttccctcgatgcgcccattgtcgcacatgaaaactatcaatgggctaactacgttcgtggcgtggtgaaacatctgcaactgcgtaacaacagcttcggcggcgtggacatggtgatcagcggcaatgtgccgcagggtgccgggttaagttcttccgcttcactggaagtcgcggtcggaaccgtattgcagcagctttatcatctgccgctggacggcgcacaaatcgcgcttaacggtcaggaagcagaaaaccagtttgtaggctgtaactgcgggatcatggatcagctaatttccgcgctcggcaagaaagatcatgccttgctgatcgattgccgctcactggggaccaaagcagtttccatgcccaaaggtgtggctgtcgtcatcatcaacagtaacttcaaacgtaccctggttggcagcgaatacaacacccgtcgtgaacagtgcgaaaccggtgcgcgtttcttccagcagccagccctgcgtgatgtcaccattgaagagttcaacgctgttgcgcatgaactggacccgatcgtggcaaaacgcgtgcgtcatatactgactgaaaacgcccgcaccgttgaagctgccagcgcgctggagcaaggcgacctgaaacgtatgggcgagttgatggcggagtctcatgcctctatgcgcgatgatttcgaaatcaccgtgccgcaaattgacactctggtagaaatcgtcaaagctgtgattggcgacaaaggtggcgtacgcatgaccggcggcggatttggcggctgtatcgtcgcgctgatcccggaagagctggtgcctgccgtacagcaagctgtcgctgaacaatatgaagcaaaaacaggtattaaagagactttttacgtttgtaaaccatcacaaggagcaggacagtgctgaggatccactagttctagagcggccgcgaagttcctatactttctagagaataggaacttctaactaaccatacatatttaaattttcaggtgctgaagtcaagtttgaaggtgatacccttgttaatagaatcgagttaaaaggtattgattttaaagaagatggaaacattcttggacacaaattggaatacaactataactcacacaatgtatacatcatggcagacaaacaaaagaatggaatcaaagttgtaagtttaaacatgattttactaactaactaatctgatttaaattttcagaacttcaaaattagacacaacattgaagatggaagcgttcaactagcagaccattatcaacaaaatactccaattggcgatggccctgtccttttaccagacaaccattacctgtcctaccaatctgccctttcgaaagatcccaacgaaaagagagaccacatggtccttcttgagtttgtaacagctgctgggattacacatggcatggatgaactatacaaatag

------- gpd-2 intergenic region

------- 1xNLS

------- YFP

------- FRT sites

------- galK

------- artificial introns

**pBALU11: gpd-2 intergenic + CFP intron-FgF**

gctgtctcatcctactttcacctagttaactgcttgtcttaaaatctatgcttctctttagtatctaaaattttcctagaagcttacaagtatataaatggtctcttctcaataaaggttgtatatttattcatcttattgaatctgccatttcctcgtttttgcgagtttatataccttccaattttctttctattgtattttcaacttctaattttaattcagggaaactgcttcaacgcatcatgaccgctccaaagaagaaacgcaaagtaccggtagaaaaaatgagtaaaggagaagaacttttcactggagttgtcccaattcttgttgaattagatggtgatgttaatgggcacaaattttctgtcagtggagagggtgaaggtgatgcaacatacggaaaacttacccttaaatttatttgcactactggaaaactacctgttccatgggtaagtttaaacatatatatactaactaaccctgattatttaaattttcagccaacacttgtcactactttctcatggggcgttcaatgcttctcgagatacccagatcatatgaaacagcatgactttttcaagagtgccatgcccgaaggttatgtacaggaaagaactatatttttcaaagatgacgggaactacaagacacgtaagtttaaacagttcggtacgaagttcctatactttctagagaataggaacttccctgttgacaattaatcatcggcatagtatatcggcatagtataatacgacaaggtgaggaactaaacccaggaggcagatcatgagtctgaaagaaaaaacacaatctctgtttgccaacgcatttggctaccctgccactcacaccattcaggcgcctggccgcgtgaatttgattggtgaacacaccgactacaacgacggtttcgttctgccctgcgcgattgattatcaaaccgtgatcagttgtgcaccacgcgatgaccgtaaagttcgcgtgatggcagccgattatgaaaatcagctcgacgagttttccctcgatgcgcccattgtcgcacatgaaaactatcaatgggctaactacgttcgtggcgtggtgaaacatctgcaactgcgtaacaacagcttcggcggcgtggacatggtgatcagcggcaatgtgccgcagggtgccgggttaagttcttccgcttcactggaagtcgcggtcggaaccgtattgcagcagctttatcatctgccgctggacggcgcacaaatcgcgcttaacggtcaggaagcagaaaaccagtttgtaggctgtaactgcgggatcatggatcagctaatttccgcgctcggcaagaaagatcatgccttgctgatcgattgccgctcactggggaccaaagcagtttccatgcccaaaggtgtggctgtcgtcatcatcaacagtaacttcaaacgtaccctggttggcagcgaatacaacacccgtcgtgaacagtgcgaaaccggtgcgcgtttcttccagcagccagccctgcgtgatgtcaccattgaagagttcaacgctgttgcgcatgaactggacccgatcgtggcaaaacgcgtgcgtcatatactgactgaaaacgcccgcaccgttgaagctgccagcgcgctggagcaaggcgacctgaaacgtatgggcgagttgatggcggagtctcatgcctctatgcgcgatgatttcgaaatcaccgtgccgcaaattgacactctggtagaaatcgtcaaagctgtgattggcgacaaaggtggcgtacgcatgaccggcggcggatttggcggctgtatcgtcgcgctgatcccggaagagctggtgcctgccgtacagcaagctgtcgctgaacaatatgaagcaaaaacaggtattaaagagactttttacgtttgtaaaccatcacaaggagcaggacagtgctgaggatccactagttctagagcggccgcgaagttcctatactttctagagaataggaacttctaactaaccatacatatttaaattttcaggtgctgaagtcaagtttgaaggtgatacccttgttaatagaatcgagttaaaaggtattgattttaaagaagatggaaacattcttggacacaaattggaatacaactatatttcacacaacgtatacatcactgccgacaaacaaaagaatgggatcaaagcggtaagtttaaacatgattttactaactaactaatctgatttaaattttcagaacttcaaaattagacacaacattgaagatggaagcgttcaactagcagaccattatcaacaaaatactccaattggcgatggccctgtccttttaccagacaaccattacctgtccacacaatctgccctttcgaaagatcccaacgaaaagagagaccacatggtccttcttgagtttgtaacagctgctgggattacacatggcatggatgaactatacaaatag

------- gpd-2 intergenic region

------- 1xNLS

------- CFP

------- FRT sites

------- galK

------- artificial introns

**pBALU12: gpd-2 intergenic + ChOpti-FgF**

gctgtctcatcctactttcacctagttaactgcttgtcttaaaatctatgcttctctttagtatctaaaattttcctagaagcttacaagtatataaatggtctcttctcaataaaggttgtatatttattcatcttattgaatctgccatttcctcgtttttgcgagtttatataccttccaattttctttctattgtattttcaacttctaattttaattcagggaaactgcttcaacgcatcatgaccgctccaaagaagaaacgcaaagtaccggtagaaaaaatggtctcaaagggtgaagaagataacatggcaattattaaagagtttatgcgtttcaaggtgcatatggagggatctgtcaatgggcatgagtttgaaattgaaggtgaaggagaaggccgaccatatgagggaacacaaaccgcaaaactaaaggtaagtttaaacatatatatactaactaaccctgattatttaaattttcaggtaactaaaggcggaccattaccattcgcctgggacatcctctctccacagttcatgtatggaagtaaagcttatgttaaacatccggcagatataccagattatttgaaactttcattcccggagggttttaagtgggaacgcgtaatgaattttgaagacggaggagttgttacagtgacgcaagactcaaggtaagtttaaacagttcggtacgaagttcctatactttctagagaataggaacttccctgttgacaattaatcatcggcatagtatatcggcatagtataatacgacaaggtgaggaactaaacccaggaggcagatcatgagtctgaaagaaaaaacacaatctctgtttgccaacgcatttggctaccctgccactcacaccattcaggcgcctggccgcgtgaatttgattggtgaacacaccgactacaacgacggtttcgttctgccctgcgcgattgattatcaaaccgtgatcagttgtgcaccacgcgatgaccgtaaagttcgcgtgatggcagccgattatgaaaatcagctcgacgagttttccctcgatgcgcccattgtcgcacatgaaaactatcaatgggctaactacgttcgtggcgtggtgaaacatctgcaactgcgtaacaacagcttcggcggcgtggacatggtgatcagcggcaatgtgccgcagggtgccgggttaagttcttccgcttcactggaagtcgcggtcggaaccgtattgcagcagctttatcatctgccgctggacggcgcacaaatcgcgcttaacggtcaggaagcagaaaaccagtttgtaggctgtaactgcgggatcatggatcagctaatttccgcgctcggcaagaaagatcatgccttgctgatcgattgccgctcactggggaccaaagcagtttccatgcccaaaggtgtggctgtcgtcatcatcaacagtaacttcaaacgtaccctggttggcagcgaatacaacacccgtcgtgaacagtgcgaaaccggtgcgcgtttcttccagcagccagccctgcgtgatgtcaccattgaagagttcaacgctgttgcgcatgaactggacccgatcgtggcaaaacgcgtgcgtcatatactgactgaaaacgcccgcaccgttgaagctgccagcgcgctggagcaaggcgacctgaaacgtatgggcgagttgatggcggagtctcatgcctctatgcgcgatgatttcgaaatcaccgtgccgcaaattgacactctggtagaaatcgtcaaagctgtgattggcgacaaaggtggcgtacgcatgaccggcggcggatttggcggctgtatcgtcgcgctgatcccggaagagctggtgcctgccgtacagcaagctgtcgctgaacaatatgaagcaaaaacaggtattaaagagactttttacgtttgtaaaccatcacaaggagcaggacagtgctgaggatccactagttctagagcggccgcgaagttcctatactttctagagaataggaacttctaactaaccatacatatttaaattttcagcctccaagatggagaatttatttataaagtcaaacttcgaggaacgaatttcccctcggatggacctgttatgcagaagaagactatgggatgggaagcttcaagtgaaagaatgtaccctgaagacggtgctcttaagggagagattaaacaacgtcttaaattgaaagatggaggacattacgatgctgaggtaagtttaaacatgattttactaactaactaatctgatttaaattttcaggtgaagacaacttacaaagccaaaaaaccagttcagctgccaggagcgtacaatgttaatattaaactggatatcacctcccacaacgaggattacactatcgttgagcaatatgaaagagctgaagggcggcactcgacaggtggcatggatgaattgtataagtaa

------- gpd-2 intergenic region

------- 1xNLS

------- mChOpti

------- FRT sites

------- galK

------- artificial introns

**pBALU13/13*: gpd-2 intergenic + FgF-2FLAG-Venus**

gctgtctcatcctactttcacctagttaactgcttgtcttaaaatctatgcttctctttagtatctaaaattttcctagaagcttacaagtatataaatggtctcttctcaataaaggttgtatatttattcatcttattgaatctgccatttcctcgtttttgcgagtttatataccttccaattttctttctattgtattttcaacttctaattttaattcagggaaactgcttcaacgcatcatgaccgctccaaagaagaaacgcaaagtaccggtagaaaaagggaagttcctatactttctagagaataggaacttccctgttgacaattaatcatcggcatagtatatcggcatagtataatacgacaaggtgaggaactaaacccaggaggcagatcatgagtctgaaagaaaaaacacaatctctgtttgccaacgcatttggctaccctgccactcacaccattcaggcgcctggccgcgtgaatttgattggtgaacacaccgactacaacgacggtttcgttctgccctgcgcgattgattatcaaaccgtgatcagttgtgcaccacgcgatgaccgtaaagttcgcgtgatggcagccgattatgaaaatcagctcgacgagttttccctcgatgcgcccattgtcgcacatgaaaactatcaatgggctaactacgttcgtggcgtggtgaaacatctgcaactgcgtaacaacagcttcggcggcgtggacatggtgatcagcggcaatgtgccgcagggtgccgggttaagttcttccgcttcactggaagtcgcggtcggaaccgtattgcagcagctttatcatctgccgctggacggcgcacaaatcgcgcttaacggtcaggaagcagaaaaccagtttgtaggctgtaactgcgggatcatggatcagctaatttccgcgctcggcaagaaagatcatgccttgctgatcgattgccgctcactggggaccaaagcagtttccatgcccaaaggtgtggctgtcgtcatcatcaacagtaacttcaaacgtaccctggttggcagcgaatacaacacccgtcgtgaacagtgcgaaaccggtgcgcgtttcttccagcagccagccctgcgtgatgtcaccattgaagagttcaacgctgttgcgcatgaactggacccgatcgtggcaaaacgcgtgcgtcatatactgactgaaaacgcccgcaccgttgaagctgccagcgcgctggagcaaggcgacctgaaacgtatgggcgagttgatggcggagtctcatgcctctatgcgcgatgatttcgaaatcaccgtgccgcaaattgacactctggtagaaatcgtcaaagctgtgattggcgacaaaggtggcgtacgcatgaccggcggcggatttggcggctgtatcgtcgcgctgatcccggaagagctggtgcctgccgtacagcaagctgtcgctgaacaatatgaagcaaaaacaggtattaaagagactttttacgtttgtaaaccatcacaaggagcaggacagtgctgaggatccactagttctagagcggccgcgaagttcctatactttctagagaataggaacttcgactacaaggacgacgacgacaagggaggagactataaggatgacgatgacaaatctggaggaggaggatctatggtgagcaagggcgaggagctgttcaccggggtggtgcccatcctggtcgagctggacggcgacgtaaacggccacaagttcagcgtgtccggcgagggcgagggcgatgccacctacggcaagctgaccctgaagctgatctgcaccaccggcaagctgcccgtgccctggcccaccctcgtgaccaccctgggctacggcctgcagtgcttcgcccgctaccccgaccacatgaagcagcacgacttcttcaagtccgccatgcccgaaggctacgtccaggagcgcaccatcttcttcaaggacgacggcaactacaagacccgcgccgaggtgaagttcgagggcgacaccctggtgaaccgcatcgagctgaagggcatcgacttcaaggaggacggcaacatcctggggcacaagctggagtacaactacaacagccacaacgtctatatcaccgccgacaagcagaagaacggcatcaaggccaacttcaagatccgccacaacatcgaggacggcggcgtgcagctcgccgaccactaccagcagaacacccccatcggcgacggccccgtgctgctgcccgacaaccactacctgagctaccagtccgccctgagcaaagaccccaacgagaagcgcgatcacatggtcctgctggagttcgtgaccgccgccgggatcactctcggcatggacgagctgtacaagtaa

------- gpd-2 intergenic region

------- 1xNLS

------- important for “frame”

------- FRT sites

------- galK

------- 2xFLAG

------- VENUS

**pBALU14/14*: gpd-2 intergenic + FgF-mCherry**

gctgtctcatcctactttcacctagttaactgcttgtcttaaaatctatgcttctctttagtatctaaaattttcctagaagcttacaagtatataaatggtctcttctcaataaaggttgtatatttattcatcttattgaatctgccatttcctcgtttttgcgagtttatataccttccaattttctttctattgtattttcaacttctaattttaattcagggaaactgcttcaacgcatcatgaccgctccaaagaagaaacgcaaagtaccggtagaaaaagggaagttcctatactttctagagaataggaacttccctgttgacaattaatcatcggcatagtatatcggcatagtataatacgacaaggtgaggaactaaacccaggaggcagatcatgagtctgaaagaaaaaacacaatctctgtttgccaacgcatttggctaccctgccactcacaccattcaggcgcctggccgcgtgaatttgattggtgaacacaccgactacaacgacggtttcgttctgccctgcgcgattgattatcaaaccgtgatcagttgtgcaccacgcgatgaccgtaaagttcgcgtgatggcagccgattatgaaaatcagctcgacgagttttccctcgatgcgcccattgtcgcacatgaaaactatcaatgggctaactacgttcgtggcgtggtgaaacatctgcaactgcgtaacaacagcttcggcggcgtggacatggtgatcagcggcaatgtgccgcagggtgccgggttaagttcttccgcttcactggaagtcgcggtcggaaccgtattgcagcagctttatcatctgccgctggacggcgcacaaatcgcgcttaacggtcaggaagcagaaaaccagtttgtaggctgtaactgcgggatcatggatcagctaatttccgcgctcggcaagaaagatcatgccttgctgatcgattgccgctcactggggaccaaagcagtttccatgcccaaaggtgtggctgtcgtcatcatcaacagtaacttcaaacgtaccctggttggcagcgaatacaacacccgtcgtgaacagtgcgaaaccggtgcgcgtttcttccagcagccagccctgcgtgatgtcaccattgaagagttcaacgctgttgcgcatgaactggacccgatcgtggcaaaacgcgtgcgtcatatactgactgaaaacgcccgcaccgttgaagctgccagcgcgctggagcaaggcgacctgaaacgtatgggcgagttgatggcggagtctcatgcctctatgcgcgatgatttcgaaatcaccgtgccgcaaattgacactctggtagaaatcgtcaaagctgtgattggcgacaaaggtggcgtacgcatgaccggcggcggatttggcggctgtatcgtcgcgctgatcccggaagagctggtgcctgccgtacagcaagctgtcgctgaacaatatgaagcaaaaacaggtattaaagagactttttacgtttgtaaaccatcacaaggagcaggacagtgctgaggatccactagttctagagcggccgcgaagttcctatactttctagagaataggaacttcgtgagcaagggcgaggaggataacatggccatcatcaaggagttcatgcgcttcaaggtgcacatggagggctccgtgaacggccacgagttcgagatcgagggcgagggcgagggccgcccctacgagggcacccagaccgccaagctgaaggtgaccaagggtggccccctgcccttcgcctgggacatcctgtcccctcagttcatgtacggctccaaggcctacgtgaagcaccccgccgacatccccgactacttgaagctgtccttccccgagggcttcaagtgggagcgcgtgatgaacttcgaggacggcggcgtggtgaccgtgacccaggactcctccctgcaggacggcgagttcatctacaaggtgaagctgcgcggcaccaacttcccctccgacggccccgtaatgcagaagaagaccatgggctgggaggcctcctccgagcggatgtaccccgaggacggcgccctgaagggcgagatcaagcagaggctgaagctgaaggacggcggccactacgacgctgaggtcaagaccacctacaaggccaagaagcccgtgcagctgcccggcgcctacaacgtcaacatcaagttggacatcacctcccacaacgaggactacaccatcgtggaacagtacgaacgcgccgagggccgccactccaccggcggcatggacgagctgtacaagtaa

------- gpd-2 intergenic region

------- 1xNLS

------- important for “frame”

------- FRT sites

------- galK

------- mCherry

**pBALU15: rla-1 cassette + GFP_intron-FgF**

gccatgttgttaccttgtattaaaacaatgtttcaagttgaatttgttcttgttgttgaattctattcacatggtctttacccatgcttcatatcttattgttttcttaccctggtcactttgtggcattaatactttctttcagcgttctatcaaacgtttgttctctttggagctaaggcaaaccgcgcaacttacgattgaagaatgaccgctccaaagaagaaacgcaaagtaccggtagaaaaaatgagtaaaggagaagaacttttcactggagttgtcccaattcttgttgaattagatggtgatgttaatgggcacaaattttctgtcagtggagagggtgaaggtgatgcaacatacggaaaacttacccttaaatttatttgcactactggaaaactacctgttccatgggtaagtttaaacatatatatactaactaaccctgattatttaaattttcagccaacacttgtcactactttctgttatggtgttcaatgcttctcgagatacccagatcatatgaaacggcatgactttttcaagagtgccatgcccgaaggttatgtacaggaaagaactatatttttcaaagatgacgggaactacaagacacgtaagtttaaacagttcggtacgaagttcctatactttctagagaataggaacttccctgttgacaattaatcatcggcatagtatatcggcatagtataatacgacaaggtgaggaactaaacccaggaggcagatcatgagtctgaaagaaaaaacacaatctctgtttgccaacgcatttggctaccctgccactcacaccattcaggcgcctggccgcgtgaatttgattggtgaacacaccgactacaacgacggtttcgttctgccctgcgcgattgattatcaaaccgtgatcagttgtgcaccacgcgatgaccgtaaagttcgcgtgatggcagccgattatgaaaatcagctcgacgagttttccctcgatgcgcccattgtcgcacatgaaaactatcaatgggctaactacgttcgtggcgtggtgaaacatctgcaactgcgtaacaacagcttcggcggcgtggacatggtgatcagcggcaatgtgccgcagggtgccgggttaagttcttccgcttcactggaagtcgcggtcggaaccgtattgcagcagctttatcatctgccgctggacggcgcacaaatcgcgcttaacggtcaggaagcagaaaaccagtttgtaggctgtaactgcgggatcatggatcagctaatttccgcgctcggcaagaaagatcatgccttgctgatcgattgccgctcactggggaccaaagcagtttccatgcccaaaggtgtggctgtcgtcatcatcaacagtaacttcaaacgtaccctggttggcagcgaatacaacacccgtcgtgaacagtgcgaaaccggtgcgcgtttcttccagcagccagccctgcgtgatgtcaccattgaagagttcaacgctgttgcgcatgaactggacccgatcgtggcaaaacgcgtgcgtcatatactgactgaaaacgcccgcaccgttgaagctgccagcgcgctggagcaaggcgacctgaaacgtatgggcgagttgatggcggagtctcatgcctctatgcgcgatgatttcgaaatcaccgtgccgcaaattgacactctggtagaaatcgtcaaagctgtgattggcgacaaaggtggcgtacgcatgaccggcggcggatttggcggctgtatcgtcgcgctgatcccggaagagctggtgcctgccgtacagcaagctgtcgctgaacaatatgaagcaaaaacaggtattaaagagactttttacgtttgtaaaccatcacaaggagcaggacagtgctgaggatccactagttctagagcggccgcgaagttcctatactttctagagaataggaacttctaactaaccatacatatttaaattttcaggtgctgaagtcaagtttgaaggtgatacccttgttaatagaatcgagttaaaaggtattgattttaaagaagatggaaacattcttggacacaaattggaatacaactataactcacacaatgtatacatcatggcagacaaacaaaagaatggaatcaaagttgtaagtttaaacatgattttactaactaactaatctgatttaaattttcagaacttcaaaattagacacaacattgaagatggaagcgttcaactagcagaccattatcaacaaaatactccaattggcgatggccctgtccttttaccagacaaccattacctgtccacacaatctgccctttcgaaagatcccaacgaaaagagagaccacatggtccttcttgagtttgtaacagctgctgggattacacatggcatggatgaactatacaaatag

------- rla-1 intergenic region

------- GFP

------- FRT sites

------- galK

------- artificial introns

**pBALU16: rla-1 cassette + YFP_intron-FgF**

gccatgttgttaccttgtattaaaacaatgtttcaagttgaatttgttcttgttgttgaattctattcacatggtctttacccatgcttcatatcttattgttttcttaccctggtcactttgtggcattaatactttctttcagcgttctatcaaacgtttgttctctttggagctaaggcaaaccgcgcaacttacgattgaagaatgaccgctccaaagaagaaacgcaaagtaccggtagaaaaaatgagtaaaggagaagaacttttcactggagttgtcccaattcttgttgaattagatggtgatgttaatgggcacaaattttctgtcagtggagagggtgaaggtgatgcaacatacggaaaacttacccttaaatttatttgcactactggaaaactacctgttccatgggtaagtttaaacatatatatactaactaaccctgattatttaaattttcagccaacacttgtcactactttcggttatggtcttcaatgcttcgccagatacccagatcatatgaaacagcatgactttttcaagagtgccatgcccgaaggttatgtacaggaaagaactatatttttcaaagatgacgggaactacaagacacgtaagtttaaacagttcggtacgaagttcctatactttctagagaataggaacttccctgttgacaattaatcatcggcatagtatatcggcatagtataatacgacaaggtgaggaactaaacccaggaggcagatcatgagtctgaaagaaaaaacacaatctctgtttgccaacgcatttggctaccctgccactcacaccattcaggcgcctggccgcgtgaatttgattggtgaacacaccgactacaacgacggtttcgttctgccctgcgcgattgattatcaaaccgtgatcagttgtgcaccacgcgatgaccgtaaagttcgcgtgatggcagccgattatgaaaatcagctcgacgagttttccctcgatgcgcccattgtcgcacatgaaaactatcaatgggctaactacgttcgtggcgtggtgaaacatctgcaactgcgtaacaacagcttcggcggcgtggacatggtgatcagcggcaatgtgccgcagggtgccgggttaagttcttccgcttcactggaagtcgcggtcggaaccgtattgcagcagctttatcatctgccgctggacggcgcacaaatcgcgcttaacggtcaggaagcagaaaaccagtttgtaggctgtaactgcgggatcatggatcagctaatttccgcgctcggcaagaaagatcatgccttgctgatcgattgccgctcactggggaccaaagcagtttccatgcccaaaggtgtggctgtcgtcatcatcaacagtaacttcaaacgtaccctggttggcagcgaatacaacacccgtcgtgaacagtgcgaaaccggtgcgcgtttcttccagcagccagccctgcgtgatgtcaccattgaagagttcaacgctgttgcgcatgaactggacccgatcgtggcaaaacgcgtgcgtcatatactgactgaaaacgcccgcaccgttgaagctgccagcgcgctggagcaaggcgacctgaaacgtatgggcgagttgatggcggagtctcatgcctctatgcgcgatgatttcgaaatcaccgtgccgcaaattgacactctggtagaaatcgtcaaagctgtgattggcgacaaaggtggcgtacgcatgaccggcggcggatttggcggctgtatcgtcgcgctgatcccggaagagctggtgcctgccgtacagcaagctgtcgctgaacaatatgaagcaaaaacaggtattaaagagactttttacgtttgtaaaccatcacaaggagcaggacagtgctgaggatccactagttctagagcggccgcgaagttcctatactttctagagaataggaacttctaactaaccatacatatttaaattttcaggtgctgaagtcaagtttgaaggtgatacccttgttaatagaatcgagttaaaaggtattgattttaaagaagatggaaacattcttggacacaaattggaatacaactataactcacacaatgtatacatcatggcagacaaacaaaagaatggaatcaaagttgtaagtttaaacatgattttactaactaactaatctgatttaaattttcagaacttcaaaattagacacaacattgaagatggaagcgttcaactagcagaccattatcaacaaaatactccaattggcgatggccctgtccttttaccagacaaccattacctgtcctaccaatctgccctttcgaaagatcccaacgaaaagagagaccacatggtccttcttgagtttgtaacagctgctgggattacacatggcatggatgaactatacaaatag

------- rla-1 intergenic region

------- 1xNLS

------- YFP

------- FRT sites

------- galK

------- artificial introns

**pBALU17: rla-1 cassette + CFP_intron-FgF**

gccatgttgttaccttgtattaaaacaatgtttcaagttgaatttgttcttgttgttgaattctattcacatggtctttacccatgcttcatatcttattgttttcttaccctggtcactttgtggcattaatactttctttcagcgttctatcaaacgtttgttctctttggagctaaggcaaaccgcgcaacttacgattgaaga atgaccgctccaaagaagaaacgcaaagtaccggtagaaaaaatgagtaaaggagaagaacttttcactggagttgtcccaattcttgttgaattagatggtgatgttaatgggcacaaattttctgtcagtggagagggtgaaggtgatgcaacatacggaaaacttacccttaaatttatttgcactactggaaaactacctgttccatgggtaagtttaaacatatatatactaactaaccctgattatttaaattttcagccaacacttgtcactactttctcatggggcgttcaatgcttctcgagatacccagatcatatgaaacagcatgactttttcaagagtgccatgcccgaaggttatgtacaggaaagaactatatttttcaaagatgacgggaactacaagacacgtaagtttaaacagttcggtacgaagttcctatactttctagagaataggaacttccctgttgacaattaatcatcggcatagtatatcggcatagtataatacgacaaggtgaggaactaaacccaggaggcagatcatgagtctgaaagaaaaaacacaatctctgtttgccaacgcatttggctaccctgccactcacaccattcaggcgcctggccgcgtgaatttgattggtgaacacaccgactacaacgacggtttcgttctgccctgcgcgattgattatcaaaccgtgatcagttgtgcaccacgcgatgaccgtaaagttcgcgtgatggcagccgattatgaaaatcagctcgacgagttttccctcgatgcgcccattgtcgcacatgaaaactatcaatgggctaactacgttcgtggcgtggtgaaacatctgcaactgcgtaacaacagcttcggcggcgtggacatggtgatcagcggcaatgtgccgcagggtgccgggttaagttcttccgcttcactggaagtcgcggtcggaaccgtattgcagcagctttatcatctgccgctggacggcgcacaaatcgcgcttaacggtcaggaagcagaaaaccagtttgtaggctgtaactgcgggatcatggatcagctaatttccgcgctcggcaagaaagatcatgccttgctgatcgattgccgctcactggggaccaaagcagtttccatgcccaaaggtgtggctgtcgtcatcatcaacagtaacttcaaacgtaccctggttggcagcgaatacaacacccgtcgtgaacagtgcgaaaccggtgcgcgtttcttccagcagccagccctgcgtgatgtcaccattgaagagttcaacgctgttgcgcatgaactggacccgatcgtggcaaaacgcgtgcgtcatatactgactgaaaacgcccgcaccgttgaagctgccagcgcgctggagcaaggcgacctgaaacgtatgggcgagttgatggcggagtctcatgcctctatgcgcgatgatttcgaaatcaccgtgccgcaaattgacactctggtagaaatcgtcaaagctgtgattggcgacaaaggtggcgtacgcatgaccggcggcggatttggcggctgtatcgtcgcgctgatcccggaagagctggtgcctgccgtacagcaagctgtcgctgaacaatatgaagcaaaaacaggtattaaagagactttttacgtttgtaaaccatcacaaggagcaggacagtgctgaggatccactagttctagagcggccgcgaagttcctatactttctagagaataggaacttctaactaaccatacatatttaaattttcaggtgctgaagtcaagtttgaaggtgatacccttgttaatagaatcgagttaaaaggtattgattttaaagaagatggaaacattcttggacacaaattggaatacaactatatttcacacaacgtatacatcactgccgacaaacaaaagaatgggatcaaagcggtaagtttaaacatgattttactaactaactaatctgatttaaattttcagaacttcaaaattagacacaacattgaagatggaagcgttcaactagcagaccattatcaacaaaatactccaattggcgatggccctgtccttttaccagacaaccattacctgtccacacaatctgccctttcgaaagatcccaacgaaaagagagaccacatggtccttcttgagtttgtaacagctgctgggattacacatggcatggatgaactatacaaatag

------- rla-1 intergenic region

------- 1xNLS

------- CFP

------- FRT sites

------- galK

------- artificial introns

**pBALU18/18*:rla-1 cassette + mChOpti_intron-FgF**

gccatgttgttaccttgtattaaaacaatgtttcaagttgaatttgttcttgttgttgaattctattcacatggtctttacccatgcttcatatcttattgttttcttaccctggtcactttgtggcattaatactttctttcagcgttctatcaaacgtttgttctctttggagctaaggcaaaccgcgcaacttacgattgaaga atgaccgctccaaagaagaaacgcaaagtaccggtagaaaaaatggtctcaaagggtgaagaagataacatggcaattattaaagagtttatgcgtttcaaggtgcatatggagggatctgtcaatgggcatgagtttgaaattgaaggtgaaggagaaggccgaccatatgagggaacacaaaccgcaaaactaaaggtaagtttaaacatatatatactaactaaccctgattatttaaattttcaggtaactaaaggcggaccattaccattcgcctgggacatcctctctccacagttcatgtatggaagtaaagcttatgttaaacatccggcagatataccagattatttgaaactttcattcccggagggttttaagtgggaacgcgtaatgaattttgaagacggaggagttgttacagtgacgcaagactcaaggtaagtttaaacagttcggtacgaagttcctatactttctagagaataggaacttccctgttgacaattaatcatcggcatagtatatcggcatagtataatacgacaaggtgaggaactaaacccaggaggcagatcatgagtctgaaagaaaaaacacaatctctgtttgccaacgcatttggctaccctgccactcacaccattcaggcgcctggccgcgtgaatttgattggtgaacacaccgactacaacgacggtttcgttctgccctgcgcgattgattatcaaaccgtgatcagttgtgcaccacgcgatgaccgtaaagttcgcgtgatggcagccgattatgaaaatcagctcgacgagttttccctcgatgcgcccattgtcgcacatgaaaactatcaatgggctaactacgttcgtggcgtggtgaaacatctgcaactgcgtaacaacagcttcggcggcgtggacatggtgatcagcggcaatgtgccgcagggtgccgggttaagttcttccgcttcactggaagtcgcggtcggaaccgtattgcagcagctttatcatctgccgctggacggcgcacaaatcgcgcttaacggtcaggaagcagaaaaccagtttgtaggctgtaactgcgggatcatggatcagctaatttccgcgctcggcaagaaagatcatgccttgctgatcgattgccgctcactggggaccaaagcagtttccatgcccaaaggtgtggctgtcgtcatcatcaacagtaacttcaaacgtaccctggttggcagcgaatacaacacccgtcgtgaacagtgcgaaaccggtgcgcgtttcttccagcagccagccctgcgtgatgtcaccattgaagagttcaacgctgttgcgcatgaactggacccgatcgtggcaaaacgcgtgcgtcatatactgactgaaaacgcccgcaccgttgaagctgccagcgcgctggagcaaggcgacctgaaacgtatgggcgagttgatggcggagtctcatgcctctatgcgcgatgatttcgaaatcaccgtgccgcaaattgacactctggtagaaatcgtcaaagctgtgattggcgacaaaggtggcgtacgcatgaccggcggcggatttggcggctgtatcgtcgcgctgatcccggaagagctggtgcctgccgtacagcaagctgtcgctgaacaatatgaagcaaaaacaggtattaaagagactttttacgtttgtaaaccatcacaaggagcaggacagtgctgaggatccactagttctagagcggccgcgaagttcctatactttctagagaataggaacttctaactaaccatacatatttaaattttcagcctccaagatggagaatttatttataaagtcaaacttcgaggaacgaatttcccctcggatggacctgttatgcagaagaagactatgggatgggaagcttcaagtgaaagaatgtaccctgaagacggtgctcttaagggagagattaaacaacgtcttaaattgaaagatggaggacattacgatgctgaggtaagtttaaacatgattttactaactaactaatctgatttaaattttcaggtgaagacaacttacaaagccaaaaaaccagttcagctgccaggagcgtacaatgttaatattaaactggatatcacctcccacaacgaggattacactatcgttgagcaatatgaaagagctgaagggcggcactcgacaggtggcatggatgaattgtataagtaa

------- rla-1 intergenic region

------- 1xNLS

------- mChOpti

------- FRT sites

------- galK

------- artificial introns

**pBALU19/19*: rla-1 cassette + 2xFLAG-VENUS**

gccatgttgttaccttgtattaaaacaatgtttcaagttgaatttgttcttgttgttgaattctattcacatggtctttacccatgcttcatatcttattgttttcttaccctggtcactttgtggcattaatactttctttcagcgttctatcaaacgtttgttctctttggagctaaggcaaaccgcgcaacttacgattgaagaatgaccgctccaaagaagaaacgcaaagtaccggtagaaaaagggaagttcctatactttctagagaataggaacttccctgttgacaattaatcatcggcatagtatatcggcatagtataatacgacaaggtgaggaactaaacccaggaggcagatcatgagtctgaaagaaaaaacacaatctctgtttgccaacgcatttggctaccctgccactcacaccattcaggcgcctggccgcgtgaatttgattggtgaacacaccgactacaacgacggtttcgttctgccctgcgcgattgattatcaaaccgtgatcagttgtgcaccacgcgatgaccgtaaagttcgcgtgatggcagccgattatgaaaatcagctcgacgagttttccctcgatgcgcccattgtcgcacatgaaaactatcaatgggctaactacgttcgtggcgtggtgaaacatctgcaactgcgtaacaacagcttcggcggcgtggacatggtgatcagcggcaatgtgccgcagggtgccgggttaagttcttccgcttcactggaagtcgcggtcggaaccgtattgcagcagctttatcatctgccgctggacggcgcacaaatcgcgcttaacggtcaggaagcagaaaaccagtttgtaggctgtaactgcgggatcatggatcagctaatttccgcgctcggcaagaaagatcatgccttgctgatcgattgccgctcactggggaccaaagcagtttccatgcccaaaggtgtggctgtcgtcatcatcaacagtaacttcaaacgtaccctggttggcagcgaatacaacacccgtcgtgaacagtgcgaaaccggtgcgcgtttcttccagcagccagccctgcgtgatgtcaccattgaagagttcaacgctgttgcgcatgaactggacccgatcgtggcaaaacgcgtgcgtcatatactgactgaaaacgcccgcaccgttgaagctgccagcgcgctggagcaaggcgacctgaaacgtatgggcgagttgatggcggagtctcatgcctctatgcgcgatgatttcgaaatcaccgtgccgcaaattgacactctggtagaaatcgtcaaagctgtgattggcgacaaaggtggcgtacgcatgaccggcggcggatttggcggctgtatcgtcgcgctgatcccggaagagctggtgcctgccgtacagcaagctgtcgctgaacaatatgaagcaaaaacaggtattaaagagactttttacgtttgtaaaccatcacaaggagcaggacagtgctgaggatccactagttctagagcggccgcgaagttcctatactttctagagaataggaacttcgactacaaggacgacgacgacaagggaggagactataaggatgacgatgacaaatctggaggaggaggatctatggtgagcaagggcgaggagctgttcaccggggtggtgcccatcctggtcgagctggacggcgacgtaaacggccacaagttcagcgtgtccggcgagggcgagggcgatgccacctacggcaagctgaccctgaagctgatctgcaccaccggcaagctgcccgtgccctggcccaccctcgtgaccaccctgggctacggcctgcagtgcttcgcccgctaccccgaccacatgaagcagcacgacttcttcaagtccgccatgcccgaaggctacgtccaggagcgcaccatcttcttcaaggacgacggcaactacaagacccgcgccgaggtgaagttcgagggcgacaccctggtgaaccgcatcgagctgaagggcatcgacttcaaggaggacggcaacatcctggggcacaagctggagtacaactacaacagccacaacgtctatatcaccgccgacaagcagaagaacggcatcaaggccaacttcaagatccgccacaacatcgaggacggcggcgtgcagctcgccgaccactaccagcagaacacccccatcggcgacggccccgtgctgctgcccgacaaccactacctgagctaccagtccgccctgagcaaagaccccaacgagaagcgcgatcacatggtcctgctggagttcgtgaccgccgccgggatcactctcggcatggacgagctgtacaagtaa

------- rla-1 intergenic region

------- 1xNLS

------- important for “frame”

------- FRT sites

------- galK

------- 2xFLAG

------- VENUS

**pBALU20/20*: rla-1 cassette + mCherry**

gccatgttgttaccttgtattaaaacaatgtttcaagttgaatttgttcttgttgttgaattctattcacatggtctttacccatgcttcatatcttattgttttcttaccctggtcactttgtggcattaatactttctttcagcgttctatcaaacgtttgttctctttggagctaaggcaaaccgcgcaacttacgattgaagaatgaccgctccaaagaagaaacgcaaagtaccggtagaaaaagggaagttcctatactttctagagaataggaacttccctgttgacaattaatcatcggcatagtatatcggcatagtataatacgacaaggtgaggaactaaacccaggaggcagatcatgagtctgaaagaaaaaacacaatctctgtttgccaacgcatttggctaccctgccactcacaccattcaggcgcctggccgcgtgaatttgattggtgaacacaccgactacaacgacggtttcgttctgccctgcgcgattgattatcaaaccgtgatcagttgtgcaccacgcgatgaccgtaaagttcgcgtgatggcagccgattatgaaaatcagctcgacgagttttccctcgatgcgcccattgtcgcacatgaaaactatcaatgggctaactacgttcgtggcgtggtgaaacatctgcaactgcgtaacaacagcttcggcggcgtggacatggtgatcagcggcaatgtgccgcagggtgccgggttaagttcttccgcttcactggaagtcgcggtcggaaccgtattgcagcagctttatcatctgccgctggacggcgcacaaatcgcgcttaacggtcaggaagcagaaaaccagtttgtaggctgtaactgcgggatcatggatcagctaatttccgcgctcggcaagaaagatcatgccttgctgatcgattgccgctcactggggaccaaagcagtttccatgcccaaaggtgtggctgtcgtcatcatcaacagtaacttcaaacgtaccctggttggcagcgaatacaacacccgtcgtgaacagtgcgaaaccggtgcgcgtttcttccagcagccagccctgcgtgatgtcaccattgaagagttcaacgctgttgcgcatgaactggacccgatcgtggcaaaacgcgtgcgtcatatactgactgaaaacgcccgcaccgttgaagctgccagcgcgctggagcaaggcgacctgaaacgtatgggcgagttgatggcggagtctcatgcctctatgcgcgatgatttcgaaatcaccgtgccgcaaattgacactctggtagaaatcgtcaaagctgtgattggcgacaaaggtggcgtacgcatgaccggcggcggatttggcggctgtatcgtcgcgctgatcccggaagagctggtgcctgccgtacagcaagctgtcgctgaacaatatgaagcaaaaacaggtattaaagagactttttacgtttgtaaaccatcacaaggagcaggacagtgctgaggatccactagttctagagcggccgcgaagttcctatactttctagagaataggaacttcgtgagcaagggcgaggaggataacatggccatcatcaaggagttcatgcgcttcaaggtgcacatggagggctccgtgaacggccacgagttcgagatcgagggcgagggcgagggccgcccctacgagggcacccagaccgccaagctgaaggtgaccaagggtggccccctgcccttcgcctgggacatcctgtcccctcagttcatgtacggctccaaggcctacgtgaagcaccccgccgacatccccgactacttgaagctgtccttccccgagggcttcaagtgggagcgcgtgatgaacttcgaggacggcggcgtggtgaccgtgacccaggactcctccctgcaggacggcgagttcatctacaaggtgaagctgcgcggcaccaacttcccctccgacggccccgtaatgcagaagaagaccatgggctgggaggcctcctccgagcggatgtaccccgaggacggcgccctgaagggcgagatcaagcagaggctgaagctgaaggacggcggccactacgacgctgaggtcaagaccacctacaaggccaagaagcccgtgcagctgcccggcgcctacaacgtcaacatcaagttggacatcacctcccacaacgaggactacaccatcgtggaacagtacgaacgcgccgagggccgccactccaccggcggcatggacgagctgtacaagtaa

------- rla-1 intergenic region

------- 1xNLS

------- important for “frame”

------- FRT sites

------- galK

------- mCherry

**pBALU21/21*: unc-54 3’UTR + FgF**

gtccaattactcttcaacatccctacatgctctttctccctgtgctcccaccccctatttttgttattatcaaaaaaacttcttcttaatttctttgttttttagcttcttttaagtcacctctaacaatgaaattgtgtagattcaaaaatagaattaattcgtaataaaaagtcgaaaaaaattgtgctccctccccccattaataataattctatcccaaaatctacacaatgttctgtgtacacttcttatgttttttttacttctgataaattttttttgaaacatcatagaaaaaaccgcacacaaaataccttatcatatgttacgtttcagtttatgaccgcaatttttatttcttcgcacgtctgggcctctcatgacgtcaaatcatgctcatcgtgaaaaagttttggagtatttttggaatttttcaatcaagtgaaagtttatgaaattaattttcctgcttttgctttttgggggtttcccctattgtttgtcaagagtttcgaggacggcgtttttcttgctaaaatcacaagtattgatgagcacgatgcaagaaagatcggaagaaggtttgggtttgaggctcagtggaaggtgagtagaagttgataatttgaaagtggagtagtgtctatggggtttttgccttaaatgacagaatacattcccaatataccaaacataactgtttcgggaagttcctatactttctagagaataggaacttccctgttgacaattaatcatcggcatagtatatcggcatagtataatacgacaaggtgaggaactaaacccaggaggcagatcatgagtctgaaagaaaaaacacaatctctgtttgccaacgcatttggctaccctgccactcacaccattcaggcgcctggccgcgtgaatttgattggtgaacacaccgactacaacgacggtttcgttctgccctgcgcgattgattatcaaaccgtgatcagttgtgcaccacgcgatgaccgtaaagttcgcgtgatggcagccgattatgaaaatcagctcgacgagttttccctcgatgcgcccattgtcgcacatgaaaactatcaatgggctaactacgttcgtggcgtggtgaaacatctgcaactgcgtaacaacagcttcggcggcgtggacatggtgatcagcggcaatgtgccgcagggtgccgggttaagttcttccgcttcactggaagtcgcggtcggaaccgtattgcagcagctttatcatctgccgctggacggcgcacaaatcgcgcttaacggtcaggaagcagaaaaccagtttgtaggctgtaactgcgggatcatggatcagctaatttccgcgctcggcaagaaagatcatgccttgctgatcgattgccgctcactggggaccaaagcagtttccatgcccaaaggtgtggctgtcgtcatcatcaacagtaacttcaaacgtaccctggttggcagcgaatacaacacccgtcgtgaacagtgcgaaaccggtgcgcgtttcttccagcagccagccctgcgtgatgtcaccattgaagagttcaacgctgttgcgcatgaactggacccgatcgtggcaaaacgcgtgcgtcatatactgactgaaaacgcccgcaccgttgaagctgccagcgcgctggagcaaggcgacctgaaacgtatgggcgagttgatggcggagtctcatgcctctatgcgcgatgatttcgaaatcaccgtgccgcaaattgacactctggtagaaatcgtcaaagctgtgattggcgacaaaggtggcgtacgcatgaccggcggcggatttggcggctgtatcgtcgcgctgatcccggaagagctggtgcctgccgtacagcaagctgtcgctgaacaatatgaagcaaaaacaggtattaaagagactttttacgtttgtaaaccatcacaaggagcaggacagtgctgaggatccactagttctagagcggccgcgaagttcctatactttctagagaataggaacttc

------- unc-54 3’UTR

------- FRT sites

------- galK

**pBALU-ext: Kan + FOSarms**

**gccagggttttcccagtcacgacgttgtaaaacgacggccagtgaattgtaatacgactcactatagggcgaattcgagctcggtacccggggatcccac**ccggaattgccagctggggcgccctctggtaaggttgggaagccctgcaaagtaaactggatggctttcttgccgccaaggatctgatggcgcaggggatcaagatctgatcaagagacaggatgaggatcgtttcgcatgattgaacaagatggattgcacgcaggttctccggccgcttgggtggagaggctattcggctatgactgggcacaacagacaatcggctgctctgatgccgccgtgttccggctgtcagcgcaggggcgcccggttctttttgtcaagaccgacctgtccggtgccctgaatgaactgcaggacgaggcagcgcggctatcgtggctggccacgacgggcgttccttgcgcagctgtgctcgacgttgtcactgaagcgggaagggactggctgctattgggcgaagtgccggggcaggatctcctgtcatcccaccttgctcctgccgagaaagtatccatcatggctgatgcaatgcggcggctgcatacgcttgatccggctacctgcccattcgaccaccaagcgaaacatcgcatcgagcgagcacgtactcggatggaagccggtcttgtcgatcaggatgatctggacgaagagcatcaggggctcgcgccagccgaactgttcgccaggctcaaggcgcgcatgcccgacggcgaggatctcgtcgtgacccatggcgatgcctgcttgccgaatatcatggtggaaaatggccgcttttctggattcatcgactgtggccggctgggtgtggcggaccgctatcaggacatagcgttggctacccgtgatattgctgaagagcttggcggcgaatgggctgaccgcttcctcgtgctttacggtatcgccgctcccgattcgcagcgcatcgccttctatcgccttcttgacgagttcttctgaattgaaaaaggaagagt**gtgggatcctctagagtcgacctgcaggcatgcaagcttgagtattctatagtctcacctaaatagcttggcgtaatcatggtcatagctgtttcctgtg**

**gccaggg…** homology to 5’ of pCC1FOS

------- Kanamycin resistance gene

**…tttcctgtg** homology to 3’ of pCC1FOS

**pCC1FOS (Epicentre)**

5’ gcggccgcaaggggttcgcgtcagcgggtgttggcgggtgtcggggctggcttaactatgcggcatcagagcagattgtactgagagtgcaccatatgcggtgtgaaataccgcacagatgcgtaaggagaaaataccgcatcaggcgccattcgccattcagctgcgcaactgttgggaagggcgatcggtgcgggcctcttcgctattacgccagctggcgaaagggggatgtgctgcaaggcgattaagttgggtaacgccagggttttcccagtcacgacgttgtaaaacgacggccagtgaattgtaatacgactcactatagggcgaattcgagctcggtacccggggatcccacgtgggatcctctagagtcgacctgcaggcatgcaagcttgagtattctatagtctcacctaaatagcttggcgtaatcatggtcatagctgtttcctgtgtgaaattgttatccgctcacaattccacacaacatacgagccggaagcataaagtgtaaagcctggggtgcctaatgagtgagctaactcacattaattgcgttgcgctcactgcccgctttccagtcgggaaacctgtcgtgccagctgcattaatgaatcggccaacgcgaaccccttgcggccgcccgggccgtcgaccaattctcatgtttgacagcttatcatcgaatttctgccattcatccgcttattatcacttattcaggcgtagcaaccaggcgtttaagggcaccaataactgccttaaaaaaattacgccccgccctgccactcatcgcagtactgttgtaattcattaagcattctgccgacatggaagccatcacaaacggcatgatgaacctgaatcgccagcggcatcagcaccttgtcgccttgcgtataatatttgcccatggtgaaaacgggggcgaagaagttgtccatattggccacgtttaaatcaaaactggtgaaactcacccagggattggctgagacgaaaaacatattctcaataaaccctttagggaaataggccaggttttcaccgtaacacgccacatcttgcgaatatatgtgtagaaactgccggaaatcgtcgtggtattcactccagagcgatgaaaacgtttcagtttgctcatggaaaacggtgtaacaagggtgaacactatcccatatcaccagctcaccgtctttcattgccatacgaaattccggatgagcattcatcaggcgggcaagaatgtgaataaaggccggataaaacttgtgcttatttttctttacggtctttaaaaaggccgtaatatccagctgaacggtctggttataggtacattgagcaactgactgaaatgcctcaaaatgttctttacgatgccattgggatatatcaacggtggtatatccagtgatttttttctccattttagcttccttagctcctgaaaatctcgataactcaaaaaatacgcccggtagtgatcttatttcattatggtgaaagttggaacctcttacgtgccgatcaacgtctcattttcgccaaaagttggcccagggcttcccggtatcaacagggacaccaggatttatttattctgcgaagtgatcttccgtcacaggtatttattcgcgataagctcatggagcggcgtaaccgtcgcacaggaaggacagagaaagcgcggatctgggaagtgacggacagaacggtcaggacctggattggggaggcggttgccgccgctgctgctgacggtgtgacgttctctgttccggtcacaccacatacgttccgccattcctatgcgatgcacatgctgtatgccggtataccgctgaaagttctgcaaagcctgatgggacataagtccatcagttcaacggaagtctacacgaaggtttttgcgctggatgtggctgcccggcaccgggtgcagtttgcgatgccggagtctgatgcggttgcgatgctgaaacaattatcctgagaataaatgccttggcctttatatggaaatgtggaactgagtggatatgctgtttttgtctgttaaacagagaagctggctgttatccactgagaagcgaacgaaacagtcgggaaaatctcccattatcgtagagatccgcattattaatctcaggagcctgtgtagcgtttataggaagtagtgttctgtcatgatgcctgcaagcggtaacgaaaacgatttgaatatgccttcaggaacaatagaaatcttcgtgcggtgttacgttgaagtggagcggattatgtcagcaatggacagaacaacctaatgaacacagaaccatgatgtggtctgtccttttacagccagtagtgctcgccgcagtcgagcgacagggcgaagccctcggctggttgccctcgccgctgggctggcggccgtctatggccctgcaaacgcgccagaaacgccgtcgaagccgtgtgcgagacaccgcggccggccgccggcgttgtggatacctcgcggaaaacttggccctcactgacagatgaggggcggacgttgacacttgaggggccgactcacccggcgcggcgttgacagatgaggggcaggctcgatttcggccggcgacgtggagctggccagcctcgcaaatcggcgaaaacgcctgattttacgcgagtttcccacagatgatgtggacaagcctggggataagtgccctgcggtattgacacttgaggggcgcgactactgacagatgaggggcgcgatccttgacacttgaggggcagagtgctgacagatgaggggcgcacctattgacatttgaggggctgtccacaggcagaaaatccagcatttgcaagggtttccgcccgtttttcggccaccgctaacctgtcttttaacctgcttttaaaccaatatttataaaccttgtttttaaccagggctgcgccctgtgcgcgtgaccgcgcacgccgaaggggggtgcccccccttctcgaaccctcccggtcgagtgagcgaggaagcaccagggaacagcacttatatattctgcttacacacgatgcctgaaaaaacttcccttggggttatccacttatccacggggatatttttataattattttttttatagtttttagatcttcttttttagagcgccttgtaggcctttatccatgctggttctagagaaggtgttgtgacaaattgccctttcagtgtgacaaatcaccctcaaatgacagtcctgtctgtgacaaattgcccttaaccctgtgacaaattgccctcagaagaagctgttttttcacaaagttatccctgcttattgactcttttttatttagtgtgacaatctaaaaacttgtcacacttcacatggatctgtcatggcggaaacagcggttatcaatcacaagaaacgtaaaaatagcccgcgaatcgtccagtcaaacgacctcactgaggcggcatatagtctctcccgggatcaaaaacgtatgctgtatctgttcgttgaccagatcagaaaatctgatggcaccctacaggaacatgacggtatctgcgagatccatgttgctaaatatgctgaaatattcggattgacctctgcggaagccagtaaggatatacggcaggcattgaagagtttcgcggggaaggaagtggttttttatcgccctgaagaggatgccggcgatgaaaaaggctatgaatcttttccttggtttatcaaacgtgcgcacagtccatccagagggctttacagtgtacatatcaacccatatctcattcccttctttatcgggttacagaaccggtttacgcagtttcggcttagtgaaacaaaagaaatcaccaatccgtatgccatgcgtttatacgaatccctgtgtcagtatcgtaagccggatggctcaggcatcgtctctctgaaaatcgactggatcatagagcgttaccagctgcctcaaagttaccagcgtatgcctgacttccgccgccgcttcctgcaggtctgtgttaatgagatcaacagcagaactccaatgcgcctctcatacattgagaaaaagaaaggccgccagacgactcatatcgtattttccttccgcgatatcacttccatgacgacaggatagtctgagggttatctgtcacagatttgagggtggttcgtcacatttgttctgacctactgagggtaatttgtcacagttttgctgtttccttcagcctgcatggattttctcatactttttgaactgtaatttttaaggaagccaaatttgagggcagtttgtcacagttgatttccttctctttcccttcgtcatgtgacctgatatcgggggttagttcgtcatcattgatgagggttgattatcacagtttattactctgaattggctatccgcgtgtgtacctctacctggagtttttcccacggtggatatttcttcttgcgctgagcgtaagagctatctgacagaacagttcttctttgcttcctcgccagttcgctcgctatgctcggttacacggctgcggcgagcgctagtgataataagtgactgaggtatgtgctcttcttatctccttttgtagtgttgctcttattttaaacaactttgcggttttttgatgactttgcgattttgttgttgctttgcagtaaattgcaagatttaataaaaaaacgcaaagcaatgattaaaggatgttcagaatgaaactcatggaaacacttaaccagtgcataaacgctggtcatgaaatgacgaaggctatcgccattgcacagtttaatgatgacagcccggaagcgaggaaaataacccggcgctggagaataggtgaagcagcggatttagttggggtttcttctcaggctatcagagatgccgagaaagcagggcgactaccgcacccggatatggaaattcgaggacgggttgagcaacgtgttggttatacaattgaacaaattaatcatatgcgtgatgtgtttggtacgcgattgcgacgtgctgaagacgtatttccaccggtgatcggggttgctgcccataaaggtggcgtttacaaaacctcagtttctgttcatcttgctcaggatctggctctgaaggggctacgtgttttgctcgtggaaggtaacgacccccagggaacagcctcaatgtatcacggatgggtaccagatcttcatattcatgcagaagacactctcctgcctttctatcttggggaaaaggacgatgtcacttatgcaataaagcccacttgctggccggggcttgacattattccttcctgtctggctctgcaccgtattgaaactgagttaatgggcaaatttgatgaaggtaaactgcccaccgatccacacctgatgctccgactggccattgaaactgttgctcatgactatgatgtcatagttattgacagcgcgcctaacctgggtatcggcacgattaatgtcgtatgtgctgctgatgtgctgattgttcccacgcctgctgagttgtttgactacacctccgcactgcagtttttcgatatgcttcgtgatctgctcaagaacgttgatcttaaagggttcgagcctgatgtacgtattttgcttaccaaatacagcaatagtaatggctctcagtccccgtggatggaggagcaaattcgggatgcctggggaagcatggttctaaaaaatgttgtacgtgaaacggatgaagttggtaaaggtcagatccggatgagaactgtttttgaacaggccattgatcaacgctcttcaactggtgcctggagaaatgctctttctatttgggaacctgtctgcaatgaaattttcgatcgtctgattaaaccacgctgggagattagataatgaagcgtgcgcctgttattccaaaacatacgctcaatactcaaccggttgaagatacttcgttatcgacaccagctgccccgatggtggattcgttaattgcgcgcgtaggagtaatggctcgcggtaatgccattactttgcctgtatgtggtcgggatgtgaagtttactcttgaagtgctccggggtgatagtgttgagaagacctctcgggtatggtcaggtaatgaacgtgaccaggagctgcttactgaggacgcactggatgatctcatcccttcttttctactgactggtcaacagacaccggcgttcggtcgaagagtatctggtgtcatagaaattgccgatgggagtcgccgtcgtaaagctgctgcacttaccgaaagtgattatcgtgttctggttggcgagctggatgatgagcagatggctgcattatccagattgggtaacgattatcgcccaacaagtgcttatgaacgtggtcagcgttatgcaagccgattgcagaatgaatttgctggaaatatttctgcgctggctgatgcggaaaatatttcacgtaagattattacccgctgtatcaacaccgccaaattgcctaaatcagttgttgctcttttttctcaccccggtgaactatctgcccggtcaggtgatgcacttcaaaaagcctttacagataaagaggaattacttaagcagcaggcatctaaccttcatgagcagaaaaaagctggggtgatatttgaagctgaagaagttatcactcttttaacttctgtgcttaaaacgtcatctgcatcaagaactagtttaagctcacgacatcagtttgctcctggagcgacagtattgtataagggcgataaaatggtgcttaacctggacaggtctcgtgttccaactgagtgtatagagaaaattgaggccattcttaaggaacttgaaaagccagcaccctgatgcgaccacgttttagtctacgtttatctgtctttacttaatgtcctttgttacaggccagaaagcataactggcctgaatattctctctgggcccactgttccacttgtatcgtcggtctgataatcagactgggaccacggtcccactcgtatcgtcggtctgattattagtctgggaccacggtcccactcgtatcgtcggtctgattattagtctgggaccacggtcccactcgtatcgtcggtctgataatcagactgggaccacggtcccactcgtatcgtcggtctgattattagtctgggaccatggtcccactcgtatcgtcggtctgattattagtctgggaccacggtcccactcgtatcgtcggtctgattattagtctggaaccacggtcccactcgtatcgtcggtctgattattagtctgggaccacggtcccactcgtatcgtcggtctgattattagtctgggaccacgatcccactcgtgttgtcggtctgattatcggtctgggaccacggtcccacttgtattgtcgatcagactatcagcgtgagactacgattccatcaatgcctgtcaagggcaagtattgacatgtcgtcgtaacctgtagaacggagtaacctcggtgtgcggttgtatgcctgctgtggattgctgctgtgtcctgcttatccacaacattttgcgcacggttatgtggacaaaatacctggttacccaggccgtgccggcacgttaaccgggctgcatccgatgcaagtgtgtcgctgtcgacgagctcgcgagctcggacatgaggttgccccgtattcagtgtcgctgatttgtattgtctgaagttgtttttacgttaagttgatgcagatcaattaatacgatacctgcgtcataattgattatttgacgtggtttgatggcctccacgcacgttgtgatatgtagatgataatcattatcactttacgggtcctttccggtgatccgacaggttacggggcggcgacctcgcgggttttcgctatttatgaaaattttccggtttaaggcgtttccgttcttcttcgtcataacttaatgtttttatttaaaataccctctgaaaagaaaggaaacgacaggtgctgaaagcgagctttttggcctctgtcgtttcctttctctgtttttgtccgtggaatgaacaatggaagtccgagctcatcgctaataacttcgtatagcatacattatacgaagttatattcgat

3’

------- 100bp 5’ of genomic DNA insertion site

------- 100bp 3’ of genomic DNA insertion site
