# Supplementary material for: A Toolkit and Robust Pipeline for the Generation of Fosmid-Based Reporter Genes in C. elegans
Source: PLoS One. 2009 Mar 4;4(3):e4625. doi: 10.1371/journal.pone.0004625 (PMC2649505; doi:10.1371/journal.pone.0004625)
Supplement: Table S1 — Comparison of our protocol to that of Dolphin and Hope (0.05 MB DOC) [file pone.0004625.s002.doc]

**Supplementary Table 1**: Comparison of our protocol to that of Dolphin and Hope

| **recombineered fosmid using protocol**  **“DH” (Dolphin & Hope, 2006) or “BALU” (this paper)** | **number of colonies analyzed for 2^nd^ step of recombineering** | **number of colonies**  **with correct recombineering** | **% efficiency of recombinnering** |
| --- | --- | --- | --- |
| WRM066bC03 (*che-1*)  DH (YFP)  BALU (pBALU4) | 30  4 | 13  4 | 43 %  100 % |
| WRM064bB08 (*die-1*)  DH (YFP)  BALU (pBALU5) | 200  8 | 4  7 | 2 %  88 % |
| WRM0619bH08 (*lsy-2*)  DH (YFP)  BALU (pBALU2)  BALU (pBALU3) | 100  4  4 | 0  4  4 | 0 %  100 %  100 % |
| WRM067cF11 (*cog-1*)  DH (YFP)  BALU (pBALU11)  BALU (pBALU17) | 600  6  8 | 1  6  5 | 0.2 %  100 %  63 % |
| WRM0624aC11(*lin-49*)  DH (YFP)  BALU (pBALU1)  BALU (pBALU2)  BALU (pBALU8) | 200  4  4  4 | 0  4  4  3 | 0 %  100 %  100 %  75 % |
| WRM0628nA07 (*lsy-6*)  DH (YFP)  BALU (pBALU3) | 100  6 | 0  5 | 0 %  84 % |
| WRM067cF11 (*R03C1.1*)  DH (mCherry)  BALU (pBALU7) | 100  4 | 0  3 | 0 %  75 % |
| WRM065aD03 (*lim-6*)  DH (YFP)  BALU | 200  not done | 3  not done | 2 %  not done |
| WRM0628cG09 (*ceh-36*)  DH (YFP)  BALU | 80  not done | 6  not done | 8 %  not done |
| WRM062aF03 (*fozi-1*)  DH (YFP)  BALU | 30  not done | 11  not done | 37 %  not done |
| WRM065cH10 (*snb-1*)  DH  BALU (pBALU10)  BALU (pBALU17) | not done  4  6 | not done  4  6 | not done  100 %  100 % |
| WRM0636dH02 (*rab-3*)  DH  BALU (pBALU11) | not done  4 | not done  4 | not done  100 % |
| WRM0611aF10 (*sng-1*)  DH  BALU (pBALU12) | not done  5 | not done  5 | not done  100 % |
| WRM0633aH07 (*gcy-1*)  DH  BALU (pBALU16)  BALU (pBALU11) | not done  4  4 | not done  4  4 | not done  100 %  100 % |
| WRM065cB03 (*gcy-22*)  DH  BALU (pBALUext) | not done  4 | not done  4 | not done  100 % |
| WRM064bB08rec_*die-1*^pBALU5^ (*flp-4*) ^1^  DH  BALU (pBALU18*) | not done  4 | not done  3 | not done  75% |

“WRM” indicates the fosmid name used for recombineering with one of the two protocol (“DH” for the Dolphin and Hope, 2006 protocol; “BALU” for the protocol of this paper). Note that the first 8 fosmid were recombineered by both the DH protocol and our protocol, allowing for direct comparison of the methods. A few cases were only attempted with either the DH protocol alone (3 reactions) or our protocol alone (7 reactions). A number of recombineerings were attempted with both, but only successful using our protocol (4 reactions) further illustrating the limited success rate of the DH protocol and the reliability of our protocol. When applying the DH protocol, we followed the provided protocol in utmost detail and in addition tried two different cassettes for selection and counter-selection; namely, the *rpsL-tetA(C)* cassette and the galK cassette. The “number of colonies analyzed for 2nd step of recombineering” reflect, for the DH protocol, the number of colonies that were picked from the Streptomycin containing plates to select for replacement of the *rpsL-tetA(C)* cassette by the reporter, except in the cases of *lsy-6* and *R03C1.1* where we used the galK cassette. In these cases, the colonies analyzed were those able to grow on DOG plates after the second recombination step. For the BALU protocol colonies that grew on minimal medium containing galactose and that were able to acidify the MacConkey indicator medium were analyzed for successful complete recombineering. The number of colonies with correct recombineering, as suggested by PCR analysis and integrity check, are included. In parentheses, the reporter for the DH protocol or pBALU cassette for the BALU protocol is indicated. ^1^ The last row shows the recombineering efficiency into the *flp-4* locus that is located on the same fosmid as the *die-1* locus, using the modified *FRT** containing construct pBALU18*. For this the fosmid WRM064bB08rec_*die-1*^pBALU5^ that contains the recombineered *die-1* locus with the unmodified *FRT* was used. The *die-1* locus remained fully stable during *FLP* recombinase induction indicating that the unmodified *FRT* does not cross react with the modified *FRT**.
