# Supplementary figures and images for: A Toolkit and Robust Pipeline for the Generation of Fosmid-Based Reporter Genes in C. elegans
Source: PLoS One. 2009 Mar 4;4(3):e4625. doi: 10.1371/journal.pone.0004625 (PMC2649505; doi:10.1371/journal.pone.0004625)

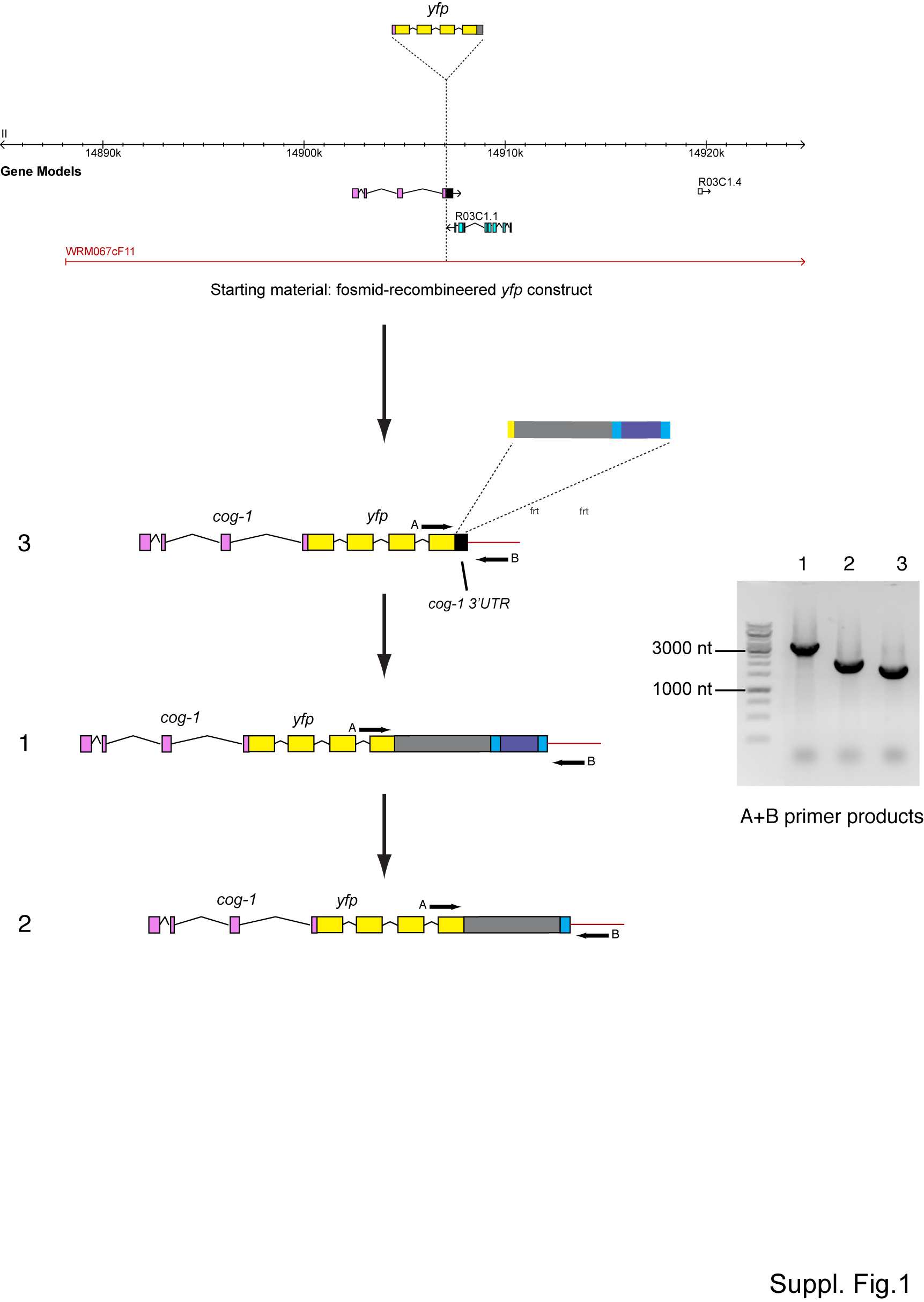

Supplement: Figure S1 — Example of sequence replacement by fosmid recombineering. The goal was to replace the miRNA-regulated 3′UTR from the cog-1 gene with that of 3′UTR from the unc-54 gene. Fosmid WRM067cF11 containing a yfp insertion in the cog-1 gene was used as the substrate for 3′ UTR replacement. pBALU 21 was used as template for amplification of the unc-54 3′ UTR fused to the FgF module flanked by homology to yfp on the 5′ side and to the gene downstream of cog-1 on the 3′ side. Recombination was performed as explained in the main text and resulted in the substitution of the full intergenic region between cog-1 and the downstream gene for the unc-54 3′ UTR and an FRT site. The agarose gel shows the products of PCR amplification with primers A and B using as template the starting fosmid (3), the fosmid with insertion of the full cassette (1) and the fosmid with the 3′ UTR replacement after excision of the FRT-galK module by Flp recombinase (2). The size of all PCR products are as expected and the final product (2) was also confirmed by sequencing. (0.55 MB TIF) [file pone.0004625.s003.tif]

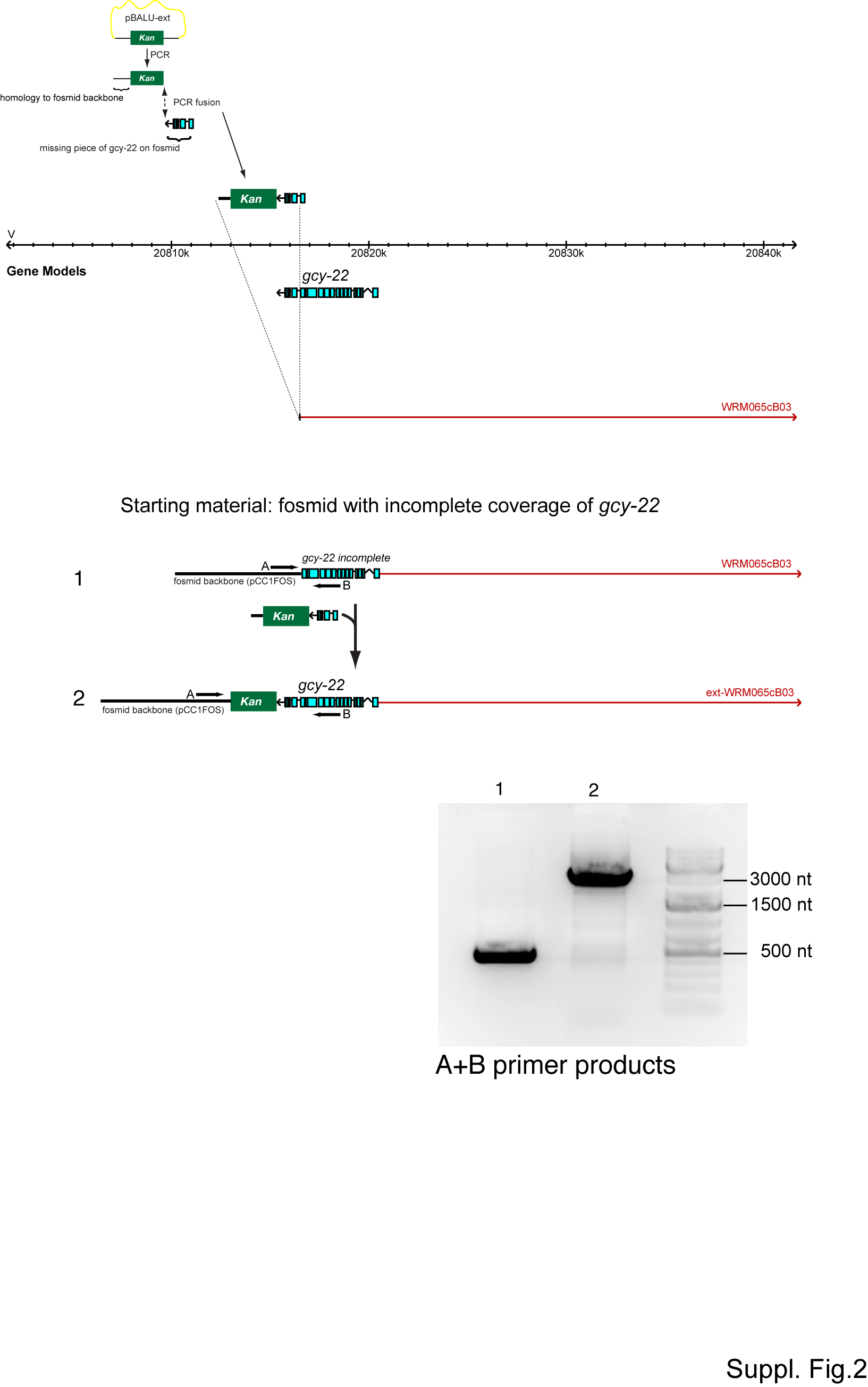

Supplement: Figure S2 — Example of extending a fosmid sequence. The goal was to extend a fosmid, WRM065cB03, to make it contain the full gcy-22 locus. This application is necessary in those relatively rare cases in which there is low fosmid coverage of a genomic region. pBALU-ext was used to amplify the kanamycin (Kan) resistance gene flanked by sequences homologous to the fosmid backbone pCC1FOS and to the genomic fragment of the gcy-22 gene that is missing from the fosmid. This gcy-22 fragment has an approx. 100 bp overlap with the incomplete gcy-22 sequence in the fosmid and was fused by PCR (Hobert, 2002) to the Kan cassette. In a single recombineering step, with subsequent Kan selection, the extension of the gcy-22 locus was accomplished with the Kan gene becoming a permanent part of the fosmid backbone. The agarose gel shows the PCR products with primers A and B that anneal to the flanking sequence on the fosmid backbone and the incomplete gcy-22. Lane 1 shows the PCR product derived from the non-extended fosmid (515 nt). Lane 2 shows the PCR product using the same primer pair after extension. The size of the PCR product corresponds with the expected size (3343 bp) after successfully completing gcy-22 with the missing fragment and adding the Kan gene to the backbone. (0.64 MB TIF) [file pone.0004625.s004.tif]
